# Determination of in vitro hepatotoxic potencies of a series of perfluoroalkyl substances (PFASs) based on gene expression changes in HepaRG liver cells

**DOI:** 10.1007/s00204-023-03450-2

**Published:** 2023-03-03

**Authors:** Jochem Louisse, Styliani Fragki, Deborah Rijkers, Aafke Janssen, Bas van Dijk, Liz Leenders, Martijn Staats, Bas Bokkers, Marco Zeilmaker, Aldert Piersma, Mirjam Luijten, Ron Hoogenboom, Ad Peijnenburg

**Affiliations:** 1 Wageningen Food Safety Research (WFSR), Wageningen, The Netherlands; 2 Centre for Health Protection, National Institute for Public Health and the Environment (RIVM), Bilthoven, The Netherlands; 3 Centre for Safety of Substances and Products, National Institute for Public Health and the Environment (RIVM), Bilthoven, The Netherlands; 4 Centre for Nutrition, Prevention and Health Services, National Institute for Public Health and the Environment (RIVM), Bilthoven, The Netherlands; 5 Institute for Risk Assessment Sciences, Utrecht University, Utrecht, The Netherlands

**Keywords:** PFASs, HepaRG cells, Transcriptomics, Relative potency, HBM4EU

## Abstract

Per- and polyfluoroalkyl substances (PFASs) are omnipresent and have been shown to induce a wide range of adverse health effects, including hepatotoxicity, developmental toxicity, and immunotoxicity. The aim of the present work was to assess whether human HepaRG liver cells can be used to obtain insight into differences in hepatotoxic potencies of a series of PFASs. Therefore, the effects of 18 PFASs on cellular triglyceride accumulation (AdipoRed assay) and gene expression (DNA microarray for PFOS and RT-qPCR for all 18 PFASs) were studied in HepaRG cells. BMDExpress analysis of the PFOS microarray data indicated that various cellular processes were affected at the gene expression level. From these data, ten genes were selected to assess the concentration–effect relationship of all 18 PFASs using RT-qPCR analysis. The AdipoRed data and the RT-qPCR data were used for the derivation of in vitro relative potencies using PROAST analysis. In vitro relative potency factors (RPFs) could be obtained for 8 PFASs (including index chemical PFOA) based on the AdipoRed data, whereas for the selected genes, in vitro RPFs could be obtained for 11–18 PFASs (including index chemical PFOA). For the readout *OAT5* expression, in vitro RPFs were obtained for all PFASs. In vitro RPFs were found to correlate in general well with each other (Spearman correlation) except for the PPAR target genes *ANGPTL4* and *PDK4*. Comparison of in vitro RPFs with RPFs obtained from in vivo studies in rats indicate that best correlations (Spearman correlation) were obtained for in vitro RPFs based on *OAT5* and *CXCL10* expression changes and external in vivo RPFs. HFPO-TA was found to be the most potent PFAS tested, being around tenfold more potent than PFOA. Altogether, it may be concluded that the HepaRG model may provide relevant data to provide insight into which PFASs are relevant regarding their hepatotoxic effects and that it can be applied as a screening tool to prioritize other PFASs for further hazard and risk assessment.

**Supplementary Information:**

The online version contains supplementary material available at 10.1007/s00204-023-03450-2.

## Introduction

Per- and polyfluoroalkyl substances (PFASs) are very persistent chemicals and omnipresent in the environment (Wang et al. 2017). PFASs are defined as “fluorinated substances that contain at least one fully fluorinated methyl or methylene carbon atom (without any H/Cl/Br/I atom attached to it)” (OECD 2021). They are widely used in various industrial and consumer applications, such as firefighting foams, electronics, textiles, food contact materials, and cosmetics. The production and use of the most studied PFASs, perfluorooctanoic acid (PFOA) and perfluorooctane sulfonate (PFOS) have been restricted given the concerns of adverse effects to human health and the environment (EU 2019, 2020; UNEP 2009).

In experimental animals, PFASs have been shown to induce a wide range of adverse effects, including hepatotoxicity, developmental toxicity, immunotoxicity, and a decrease in thyroid hormone levels (ATSDR 2021; EFSA CONTAM Panel 2018, 2020). The most consistent endpoint is increased liver weight, characterized by a combined hyperplasia and hypertrophy, which has been observed for many PFASs with clear differences in potencies. Disturbances in lipid metabolism, including hepatocellular steatosis and other hepatotoxic effects, have also been reported (EFSA CONTAM Panel 2020). Also in humans, rather low serum levels of PFOS and PFOA have been associated with disturbed lipid homeostasis, in which the liver may play a role. However, the causality of this relationship has been debated (see for a recent review Fragki et al. (2021)). Furthermore, epidemiological evidence has correlated serum levels of both PFOS and PFOA to a small elevation in serum levels of the hepatic enzyme ALT (alanine transferase), a biomarker for liver damage (Gallo et al. 2012). However, whether that limited increase in ALT reflects serious liver damage is questionable.

Bil et al. (2021, 2022a) used data on hepatotoxicity in individual studies with male rats to derive external relative potency factors (RPFs) for 16 PFASs (using PFOA as index chemical). External RPFs of 7 other PFASs were estimated based on read across. In addition, Bil et al. (2022b) reported eight internal RPFs, which are based on the same toxicological information as the external RPFs reported by Bil et al. (2021, 2022a), but estimated by translating external doses to internal blood concentrations using kinetic models. For assessment of risks upon combined exposure to PFASs, such RPFs may be of use to take potency differences in PFASs into account. In that regard, external RPFs may be of use when considering external exposure and internal RPFs when considering internal exposure.

The number of existing PFASs is estimated to be around a few thousands, and for many of these, toxicity data are lacking. Performing in vivo animal studies to obtain toxicity data for all these PFASs is not considered feasible, given the high costs and demand of resources, and also not desirable, because of ethical issues and the uncertainty related to possible species differences between laboratory animals and humans. Instead, novel approach methodologies (NAMs), such as in vitro toxicity assays, may be used, in the first place to prioritize those PFASs for which a more extensive hazard and risk assessment would be considered most relevant, and within a next-generation risk assessment paradigm, to provide in vitro effect concentrations that can be translated to in vivo oral equivalent dose levels (Punt et al. 2021), providing data that may be used for the risk assessment.

**Fig. 1:**
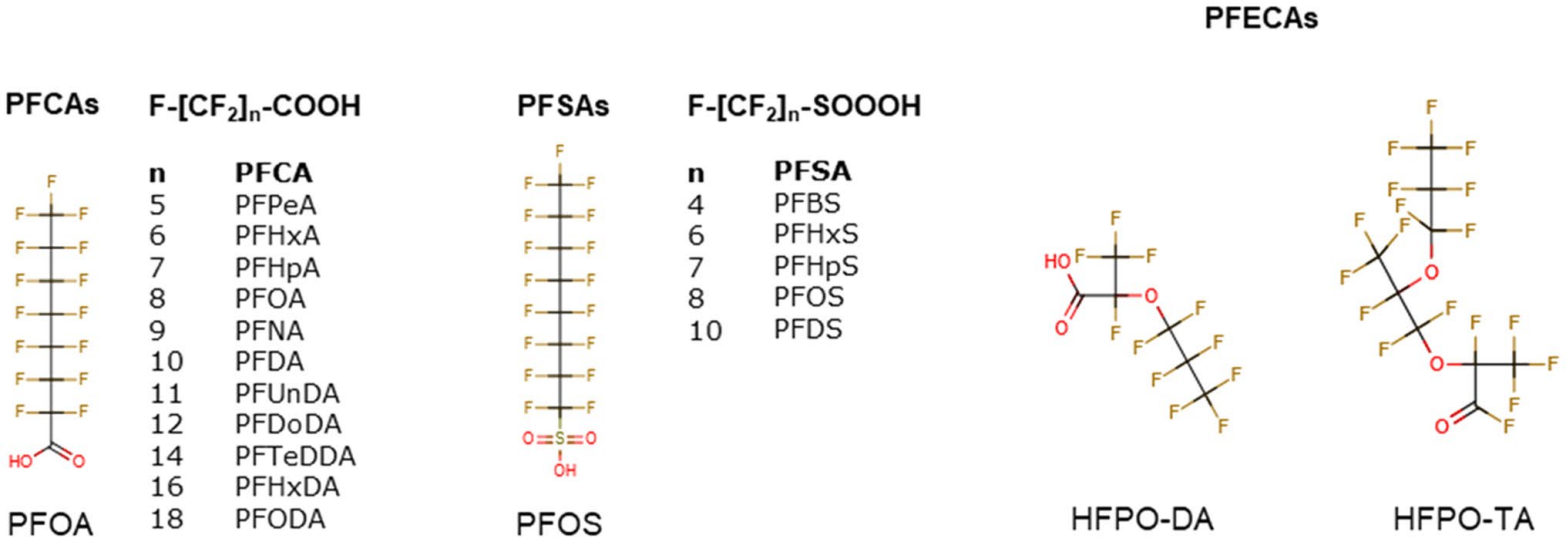
Chemical structures of the PFASs tested in the present study. Full names of abbreviations are provided in the Materials and methods section under ‘Chemicals’

Recently, we demonstrated that treatment of HepaRG human liver cells with PFOA, PFOS, and PFNA resulted in an increase in triglyceride levels (Louisse et al. 2020), which is considered to be a potential relevant readout for PFAS-induced liver toxicity (Fragki et al. 2021). Furthermore, microarray analysis indicated that these three PFASs, at a concentration of 100 μM, downregulated genes involved in cholesterol biosynthesis. The data also pointed to, among others, changes in cellular processes, such as PERK/ATF4 signaling, tRNA aminoacylation and expression of amino acid transporters by PFOA, PFOS and PFNA. It is of interest to assess whether such in vitro effects may be of use for obtaining insight into potency differences of different PFASs. Therefore, the present study aimed to assess the concentration-dependent effects of 18 PFASs (Fig. 1) on triglyceride levels (applying the AdipoRed assay) and expression of genes (as measured with RT-qPCR) in HepaRG cells. This study includes 11 perfluoroalkyl carboxylic acids (PFCAs), 5·perfluoroalkyl sulfonic acids (PFSAs) and 2 perfluoroalkyl ether carboxylic acids (PFECAs, including GenX (HFPO-DA)). To identify genes for RT-qPCR analysis, concentration-dependent PFOS transcriptomic data were analyzed with BMDExpress software, providing insight into PFOS-induced effects on gene expression and their concentration-dependency in HepaRG cells. Based on these data, genes were selected to assess the concentration-dependent changes in expression upon exposure to the 18 PFASs (Fig. 1). Concentration–response data on the increase in triglyceride levels and effects on gene expression of the selected genes were analyzed with PROAST software to obtain insight into in vitro potency differences for the 18 PFASs. The obtained in vitro RPFs were compared with reported external and internal RPFs obtained from animal studies to provide insights into differences and similarities in the outcomes of using in vitro human cell-based and in vivo animal-based approaches.

## Materials and methods

### Chemicals

The following PFASs were tested in the present study: perfluoropentanoic acid (PFPeA; C5), perfluorohexanoic acid (PFHxA; C6), perfluoroheptanoic acid (PFHpA; C7), perfluorooctanoic acid (PFOA; C8), perfluorononanoic acid (PFNA; C9), perfluorodecanoic acid (PFDA; C10), perfluoroundecanoic acid (PFUnDA; C11), perfluorododecanoic acid (PFDoDA; C12), perfluorotetradecanoic acid (PFTeDA; C14), perfluorohexadecanoic acid (PFHxDA; C16), perfluorooctadecanoic acid (PFODA; C18), perfluorobutane sulfonate (PFBS; C4), perfluorohexane sulfonate (PFHxS; C6), perfluoroheptane sulfonate (PFHpS; C7), perfluorooctane sulfonate (PFOS; C8), perfluorodecane sulfonate (PFDS; C10), hexafluoropropylene oxide dimer acid (HFPO-DA, also known as GenX; C6) and hexafluoropropylene oxide trimer acid (HFPO-TA; C9) (Fig. 1). All stocks were prepared in 100% dimethyl sulfoxide (DMSO HybriMax, Sigma-Aldrich), which were stored at – 20 °C. More information about suppliers, purity, catalog numbers, CAS numbers and maximum concentrations tested in the present study is presented in Supplementary Table 1. The highest concentration tested was determined by the degree of solubility of each PFAS.

### HepaRG cell culture

The human hepatic cell line HepaRG was obtained from Biopredic International (Rennes, France) and cultured in growth medium consisting of William’s Medium E + GlutaMAX^™^ (ThemoFisher Scientific, Landsmeer, The Netherlands) supplemented with 10% fetal bovine serum (FBS; Corning (35-079-CV), United States of America), 1% PS (100 U/mL penicillin, 100 µg/mL streptomycin; Capricorn Scientific, Ebsdorfergrund, Germany), 50 µM hydrocortisone hemisuccinate (sodium salt) (Sigma-Aldrich), and 5 µg/mL human insulin (PAN^™^ Biotech). Seeding, trypsinization (using 0.05% Trypsin–EDTA (ThermoFisher Scientific)) and maintenance of the cells was performed according to the HepaRG instruction manual from Biopredic International. For cell viability and triglyceride accumulation studies, cells were seeded in black-coated 96-well plates (Greiner Bio-One, Frickenhausen, Germany; 9000 cells per well in 100 µL). For gene expression studies, cells were seeded in 24-well plates (Corning, Corning, NY; 55,000 cells per well in 500 µL). After two weeks on growth medium, cells were cultured for two days in growth medium supplemented with 0.85% DMSO to induce differentiation. Subsequently, cells were cultured for 12 days in growth medium supplemented with 1.7% DMSO (differentiation medium) for final differentiation. At this stage, cells were ready to be used for toxicity studies. Cells that were not immediately used were kept on differentiation medium for a maximum of three additional weeks. Cell cultures were maintained in an incubator (humidified atmosphere with 5% CO_2_ at 37 °C) and the medium was refreshed every 2–3 days during culturing. Prior to toxicity studies, differentiated cells were incubated for 24 h in assay medium (growth medium containing 2% FBS) supplemented with 0.5% DMSO.

### Cell exposure

Test chemicals were diluted from 200-fold concentrated stock solutions in assay medium, providing a final DMSO concentration of 0.5%. In each experiment a solvent control (0.5% DMSO) was included. PFASs were tested in concentrations up to 400 µM (if solubility allowed). After exposure, effects of the PFASs on cell viability and gene expression were assessed. Highest tested concentrations that could be tested for each PFAS are presented in Supplementary Table 1.

### Stability studies HFPO-DA and HFPO-TA

To assess whether HFPO-DA and HFPO-TA are stable under the culture conditions applied in this study, we incubated 50 µM HFPO-DA or HFPO-TA in culture medium (0.5% DMSO) for 24 h in an incubator (humidified atmosphere with 5% CO_2_ at 37 °C) and took samples at *t* = 0 h, 6 h and 24 h for quantification using LC–MS analysis. We also assessed stability of stock solutions in DMSO kept at – 20 °C. To 50 uL culture medium, 850 uL methanol (Actuall Chemicals, Oss, The Netherlands) containing internal standard (^13^C_3_-GenX (Wellington Laboratories, Canada)) was added. These dilutions were vortexed well before centrifugation at maximum speed for 10 min at 4 °C. Samples were further another 1200 times diluted with methanol and internal standard, and HFPO-DA and HFPO-TA concentrations were determined using LC–MS/MS analysis. LC–MS/MS analysis was based on a Sciex UHPLC system containing: 2 pumps (ExionLC AD); column oven (ExionLC AC); controller (ExionLC); degasser (ExionLC); and sample tray holder (ExionLC AD) (Sciex, Framingham, MA, USA). Luna Omega PS C18 analytical column (100Å, 100 × 2.1 mm i.d., 1.6 μm, Phenomenex, Torrance, CA, USA) was used to separate the PFASs at a column temperature of 40 °C. Additionally, a Gemini C18 analytical column (110Å, 50 × 3 mm i.d., 3 µm, Phenomenex, Torrance, CA, USA) was used as an isolator column, placed between the pump and the injector valve to isolate and delay interferences out of the LC system. The mobile phase consisted of 20 mM ammonium acetate (Merck Millipore, Darmstadt, Germany) in water Ultra LC/MS grade (Actu-All Chemicals, Oss, The Netherlands) (mobile phase A) and Acetonitrile ULC/MS grade (Biosolve, Dieuze, France) (mobile phase B). The injection volume used was 20 μL. The chromatographic gradient was operated at a flow rate of 0.8 mL min^−1^ starting from 15% mobile phase B in the first 1.0 min, a linear increase to 98% B in 6 min with a final hold of 0.5 min. The gradient was returned to 15% B within 0.1 min for 0.7 min to equilibrate before the next injection, resulting in a total run of 8.3 min.

Detection was carried out by MS/MS using a Sciex QTRAP 7500 system (Sciex, Framingham, MA, USA) in negative electrospray ionization (ESI-) mode, with the following conditions: ion spray voltage (IS) of – 1500 V; curtain gas (CUR) of 45 psi; source temperature (TEM) of 400 °C; gas 1 (GS1) of 40 psi; gas 2 (GS2) of 80 psi; and collision gas (CAD) 9. The PFASs were fragmented using collision induced dissociation (CID) using argon as target gas. The analyses were performed in multiple reaction monitoring (MRM) mode, using two mass transitions per component selected based on the abundance of the signal and the selectivity of the transition. In Supplementary Table 2, information on the MRM transitions, entrance potential (EP), collision energy (CE) and cell exit potential (CXP) is presented. Data were acquired using SciexOS and processed using MultiQuantTM software (Sciex, Framingham, MA, USA).

### Cell viability studies

The effects of the 18 PFASs on the viability of HepaRG cells cultured in 96-well plates were determined using the WST-1 assay. This assay determines the conversion of the tetrazolium salt WST-1 (4-[3-(4-iodophenyl)-2-(4-nitrophenyl)-2H-5-tetrazolio]-1,3-benzene disulfonate) to formazan by metabolically active cells. For PFOA, PFNA, PFHxS and PFOS, the effects on cell viability were studied upon a 24-h and a 72-h exposure, given that both exposure times were studied for optimization of the exposure time for assessing effects of these PFASs on triglyceride accumulation. All other PFASs were only tested upon a 24-h exposure. After exposure, the medium was removed and the cells were washed with Dulbecco’s Phosphate Buffered Saline (DPBS; ThermoFisher Scientific). Next, WST-1 solution (Sigma-Aldrich) was added to the cell culture medium (1:10 dilution), and 100 µL was added to each well. After 1 h incubation in an incubator (humidified atmosphere with 5% CO_2_ at 37 °C), the plate was shaken at 1000 rpm for 1 min, and absorbance at 450 nm was measured (background absorbance at 630 nm was subtracted) using a Synergy HT Microplate Reader (BioTek, Winooski, VT). Three independent studies, with in each study three technical replicates per condition, were performed. Cell viability upon PFAS treatments was expressed as percentage of the cell viability of the solvent control.

### Triglyceride accumulation studies

The effect of the 18 PFASs on triglyceride levels was determined using the AdipoRed assay essentially according to the instructions of the supplier (Lonza, Basel, Switzerland). We used the approach as applied in the study of Luckert et al. (2018), in which HepaRG cells were exposed to the steatotic compound cyproconazole. In that study, 72 h was shown to be the optimal time point to assess the effects of cyproconazole on triglyceride accumulation as determined with the AdipoRed assay. We first assessed whether this time point was also the optimal time point for assessing effects of PFASs on triglyceride accumulation, by studying the effects of a 24-h or a 72-h exposure to PFOA, PFNA, PFHxS and PFOS in the AdipoRed assay, also including cyproconazole as positive control. After exposure for 24 or 72 h, the medium was removed and the cells were washed with 200 μL DPBS and subsequently incubated for 10 min at room temperature with 200 μL AdipoRed-DPBS solution. The latter solution was prepared by adding 25 μL AdipoRed to 1 mL DPBS. Subsequently, fluorescence was measured using a 485/20 nm excitation and 590/35 emission filter set on the Synergy HT Microplate Reader. The results from that study indicate that a 24-h exposure was considered better than a 72-h exposure to study effects of PFASs (see Results section). Therefore, all other PFASs were tested upon a 24-h exposure. For each PFAS, three independent biological replicates, with three technical replicates per condition were obtained. Data were used for dose–response analysis using PROAST software (see below).

### Whole genome gene expression: microarray hybridizations and BMDExpress analysis

To obtain insight into the PFOS concentration-dependent induced gene expression changes, differentiated cells were exposed for 24 h to 6.25, 12.5, 25, 50, 100, 200, or 400 µM PFOS. An exposure duration of 24 h was selected based on our previous study (Louisse et al. 2020). After exposure, total RNA was isolated and purified using the RNeasy Minikit (Qiagen). RNA quality and integrity was assessed using the RNA 6000 Nano chips on the Agilent 2100 Bioanalyzer (Agilent Technologies, Amsterdam, The Netherlands). Purified RNA (100 ng) was labeled with the Ambion WT expression kit (Invitrogen) and hybridized to Affymetrix Human Gene 2.1 ST arrays (Affymetrix, Santa Clara, CA). Hybridization, washing, and scanning were carried out on an Affymetrix GeneTitan platform according to the instruction by the manufacturer. Obtained data (CEL-files) were further processed using Bioconductor in R, performing quality control and normalization. For array normalization, the Robust Multiarray Average method (Bolstad et al. 2003; Irizarry et al. 2003) was applied. Probe sets were defined according to Dai et al. (2005). In this method, probes are assigned to Entrez IDs as a unique gene identifier. CEL file normalization was performed with the Robust Multichip Average method using the Bioconductor oligo package (version 3.8) and the human Entrez-Gene custom CDF annotation from Brain Array version 23.0.0 containing 965,365 probes and 29,635 probesets (http://brainarray.mbni.med.umich.edu/Brainarray/Database/CustomCDF/CDF_download.asp).

BMDExpress is a software tool for BMD analysis of transcriptomic data (Yang et al. 2007; Phillips et al. 2019). BMDExpress-2 (Version 2.20.0180) was applied following the workflow (loading expression data, filtering, BMD analysis, and Pathway analysis (functional analysis)) as described on https://github.com/auerbachs/BMDExpress-2/wiki. Expression data were organized in a tab-delimited plain text file and are provided as Supplementary Material. Each column in the data matrix corresponds to an individual expression experiment. The first row contains information in the sample label, the second row on the PFOS concentration and all further rows the data for one probe ID. Regarding loading of the expression data, ‘Generic’ was selected for the platform, and ‘BASE2’ for the Log Transformation. Regarding the filtering, ANOVA was used, using a *p* value Cutoff of 0.05, applying the Benjamin & Hochberg correction for multiple testing, filtering out control genes, and without applying a Fold Change Filter (i.e., Fold Change Value of 1.0 was selected). Regarding BMD analysis, the continuous models Exp2, Exp3, Exp4, Exp5, Linear, Poly2, Poly3, Hill and Power were selected. A BMR factor of 1.021 was selected, as also applied by Chang et al. (2020). Applying such a low response as BMR allows inclusion of genes that may show limited changes in expression. Application of a higher BMR may provide more robust BMC estimations, but may exclude genes (and as a possible consequence-related gene sets) that show limited gene expression changes that could be relevant from a biological perspective. Regarding the functional analysis, we performed a defined category analysis using gene sets from the Reactome Pathway Database (https://reactome.org/; Wu and Haw 2017), applying the following data source options: ‘Remove Promiscuous Probes’, ‘Remove BMD > Highest Dose from Category Descriptice Statistics’, ‘Remove BMD with *p* Value < Cutoff: 0.1’, ‘Remove genes with BMD/BMDL > : 20’, ‘Remove genes with BMDU/BMDL > : 40’, ‘Remove Genes With Max Fold Change < : 1.2’, and ‘Identify conflicting probe sets: 0.5’. The applied probe file and the category file used for the analysis are provided in the Supplementary Materials. For further analysis, we applied the following filters: Fisher’s Exact Two Tail ≤ 0.1, ‘genes that passed all filters’ of a gene set were set at 5, and the percentage of genes regulated of the gene was set at ≥ 20%. For the gene sets remaining upon application of these filters, information was collected and organized in an Excel file, which is available as Supplementary Material.

### RT-qPCR

For selected genes, concentration-dependent expression levels were determined in PFAS-exposed HepaRG cells. To that end, cells were exposed to increasing concentrations of the 18 PFASs for 24 h and total RNA was extracted from the cells using the RNeasy Mini Kit (Qiagen, Venlo, The Netherlands). Subsequently, 500 ng RNA was used to synthesize cDNA using the iScript cDNA synthesis kit (Bio-Rad Laboratories, Veenendaal, The Netherlands). Changes in gene expression were determined by RT-qPCR on a CFX384 real-time PCR detection system (Bio-Rad Laboratories) using SensiMix (Bioline; GC Biotech, Alphen aan den Rijn, The Netherlands). The PCR conditions consisted of an initial denaturation at 95 °C for 10 min, followed by 40 cycles of denaturation at 95 °C for 10 s and annealing extension at 60 °C for 15 s. Relative gene expression was quantified with the standard curve method, using a standard curve generated from a serial dilution of pooled sample cDNA, and subsequently normalized to *RPL27* gene expression. Primer sequences were taken from the Harvard PrimerBank and ordered from Eurogentec (Liège, Belgium). Sequences of the used primers are listed in Table 1. The concentration–response data were subjected to dose–response analysis using PROAST software as described below.

**Table 1:** Primer sequences used for RT-qPCR

Name	Primer sequence	
	Forward	Reverse
*ANGPTL4*	CACAGCCTGCAGACACAACTC	GGAGGCCAAACTGGCTTTGC
*ATF4*	CCCTTCACCTTCTTACAACCTC	TGCCCAGCTCTAAACTAAAGGA
*CXCL10*	GAACTGTACGCTGTACCTGCA	TTGATGGCCTTCGATTCTGGA
*HMGCR*	TGATTGACCTTTCCAGAGCAAG	CTAAAATTGCCATTCCACGAGC
*LSS*	GCACTGGACGGGTGATTATGG	TCTCTTCTCTGTATCCGGCTG
*OAT5*	TGGTGTTTGCTCCAGCTTG	GCCTTATCCACTCAGTAATGGGC
*PDK4*	TGGAGCATTTCTCGCGCTAC	ACAGGCAATTCTTGTCGCAAA
*RPL27*	ATCGCCAAGAGATCAAAGATAA	TCTGAAGACATCCTTATTGACG
*SLC7A11*	GGTCCATTACCAGCTTTTGTACG	AATGTAGCGTCCAAATGCCAG
*THRSP*	CAGGTGCTAACCAAGCGTTAC	CAGAAGGCTGGGGATCATCA
*YARS1*	TGGTCACACAGCACGATTCC	CGGGGTATAAGAGGCCACTC

### Dose–response analysis of AdipoRed and RT-qPCR data with PROAST

AdipoRed data and RT-qPCR data were used for concentration–response modeling with dose–response analysis software PROAST version 70.2 and 70.7tmp (National Institute for Public Health and the Environment 2018) in R (version 4.2.0). Data were available from three independent experiments (n = 3). First, it was determined whether differences between the independent experiments (for individual PFASs) exist. For this, PROAST version 70.2 was used. This analysis was performed using the data of *OAT5* gene expression. It appeared that the background (parameter *a*) differed for some PFASs between different experiments, based on which it was decided to not use summary data for the further dose–response analysis to determine RPFs, but to run the PROAST analyses (in version 70.7tmp) with the following covariates: substance (parameter *b* and var) and substance experiment (parameter *a*). Data of all PFASs were analyzed simultaneously to ensure the parallel curves required to derive RPFs (Bosgra et al. 2009; Bil et al. 2021, 2022a, b; van der Ven et al. 2022; van den Brand et al. 2022). Tab-delimited text files containing data on concentration, effect, and experiment number were made and analyzed as continuous data. Non-normalized gene expression and AdipoRed data were used for dose–response analysis since possible differences in background are accounted for by the covariate on background parameter *a*. Then, the exponential model,

$$y=a*{c}^{1-\mathrm{exp}\left(-{\left(x/b\right)}^{d}\right)}$$

with parameters *a*, *b*, *c*, and *d* describing the response at dose 0 (background value), the potency of the PFAS, maximum fold change in response compared with background response (upper or lower plateau), and steepness of the curve (on a log-dose scale), respectively, was fitted with and without fixing parameter *c* to a large value to determine if a maximum fold change could be established. The model (with or without fixed parameter *c*) with the lowest Akaike information criterion (AIC) was chosen to determine the RPFs and the corresponding 90% confidence intervals (Bil et al. 2022a, b; van den Brand et al. 2022). PFOA was used as the index chemical. For some PFASs, it was not possible to determine an RPF and for some compounds, determination of the lower bound RPF (RPFL) was not possible, because the data did not show a clear trend.

### Comparison of obtained in vitro RPFs and reported in vivo RPFs

We compared the RPFs obtained from the different in vitro readouts (AdipoRed and selected genes) to assess whether different conclusions would be drawn based on the readout selection. Subsequently we compared the in vitro RPFs with RPFs reported in the literature obtained from in vivo rat studies, for which RPFs are available for external (Bil et al. 2021, 2022a) and internal exposure (Bil et al. 2022b).

## Results

### Stability studies HFPO-DA and HFPO-TA

Since a recent study has shown that certain PFECAs, including HFPO-DA and HFPO-TA degrade when present in acetonitrile, acetone or DMSO (Zhang et al. 2022), we assessed whether HFPO-DA and HFPO-TA are stable under the culture conditions applied in this study. To that end, HFPO-DA and HFPO-TA were added to cell culture medium (0.5% DMSO) at a concentration of 50 µM and incubated in an incubator (humidified atmosphere with 5% CO_2_ at 37 °C). Samples were taken at *t* = 0 h, 6 h and 24 h, and were measured using LC–MS analysis. Results show that under these conditions, HFPO-DA and HFPO-TA are stable (Supplementary Fig. 1), indicating that these culture conditions are adequate to determine the effects of these PFASs on HepaRG cells.

### Cell viability studies

The effect of a 24-h (all PFASs) and 72-h (PFOA, PFNA, PFHxS, and PFOS) exposure of HepaRG cells to the PFASs on cell viability was determined using the WST-1 assay. Concentrations up to 400 µM were used, except for PFDoDA/ PFTeDA (up to 100 µM) and PFHxDA/PFODA (up to 25 µM), due to limited solubility of these PFASs (Supplementary Table 1). The results of the 72-h exposure studies indicate that PFOA is clearly cytotoxic at 400 µM, PFNA at 200 and 400 µM and that no effects were found for PFHxS and PFOS (Supplementary Fig. 2). The results of the 24-h exposure studies indicate that four of the 18 tested PFASs decrease cell viability in a concentration-dependent manner, being PFNA, PFDA, PFHpS and HPFO-TA (Supplementary Fig. 3), with HFPO-TA being the most potent PFAS, followed by PFDA and PFNA. The other PFASs did not show cytotoxicity in the WST-1 assay for the concentration range tested (Supplementary Fig. 3).

Maximum concentrations for the further studies were selected as the highest concentrations causing less than 25% decrease in cell-based WST-1 conversion, amounting to 50 µM HPFO-TA, 100 µM PFNA, 100 µM PFDA and 200 µM PFHpS.

### Triglyceride accumulation studies

**Fig. 2:**
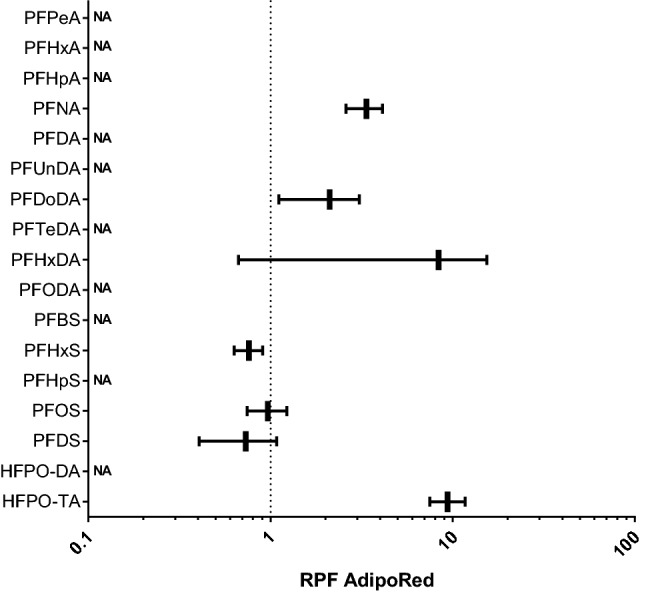
In vitro RPFs based on PROAST dose–response analysis of AdipoRed data obtained from HepaRG cells exposed to various PFASs. RPFs are presented as vertical lines, with the 5% lower bound and 95% upper bound of the confidence interval as whiskers. PFOA was used as index chemical, i.e., has an RPF of 1 (dotted line). *NA* not applicable, RPF could not be determined

We first assessed whether a 24-h or a 72-h exposure was considered optimal to assess effects of PFASs on triglyceride accumulation, as measured with the AdipoRed assay, by determining the effects for PFOA, PFNA, PFHxS and PFOS, also including cyproconazole, for which earlier studies indicated that most effects were found upon a 72-h exposure (Luckert et al. 2018). The results show that for the four PFASs, in contrast to cyproconazole, more pronounced effects were observed upon exposure for 24 h compared to a 72-h exposure (Supplementary Fig. 4). Therefore, for all other PFASs, the effect of a 24-h exposure to the PFASs on triglyceride accumulation in HepaRG cells was determined. In general, PFAS-induced changes in AdipoRed signal were relatively limited, at maximum amounting to a 1.4-fold increase at 50 µM HPFO-TA versus the solvent control (Supplementary Fig. 5), compared to a measured maximum 1.6-fold increase for the positive control cyproconazole (Supplementary Fig. 4). Dose–response analysis using parallel curve fitting was applied on the AdipoRed data to determine in vitro RPF values, which could be obtained for PFNA, PFDoDA, PFHxDA, PFHxS, PFOS, and PFDS (Fig. 2). PFNA, PFDoDA, PFHxDA, and HFPO-TA were more potent than PFOA, but it must be noted that confidence intervals of PFHxDA’s RPF are large (Fig. 2). PFOS showed a similar potency as PFOA, and PFHxS and PFDS were slightly less potent than PFOA (Fig. 2).

### Transcriptomics studies PFOS-exposed HepaRG cells and BMDExpress analysis

**Fig. 3:**
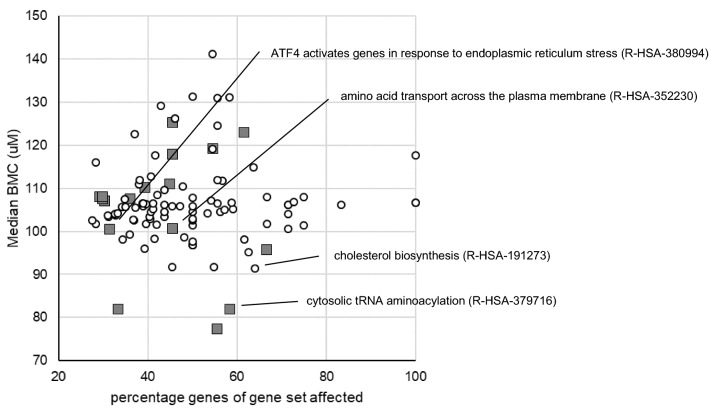
Overview of upregulated (gray squares) and downregulated (white circles) Reactome gene sets based on microarray data of PFOS-exposed HepaRG cells as analyzed with BMDExpress. Each gene set is positioned based on the percentage of affected genes of the gene set and the median BMC value of the gene set. Gene sets related to cellular processes that were previously found to be affected by PFOA, PFOS and PFNA in HepaRG cells (Louisse et al. 2020) are indicated. More information on the regulated genes of these gene sets is presented in Fig. 4. More information on all affected gene sets is presented in the Supplementary Materials (color figure online)

**Fig. 4:**
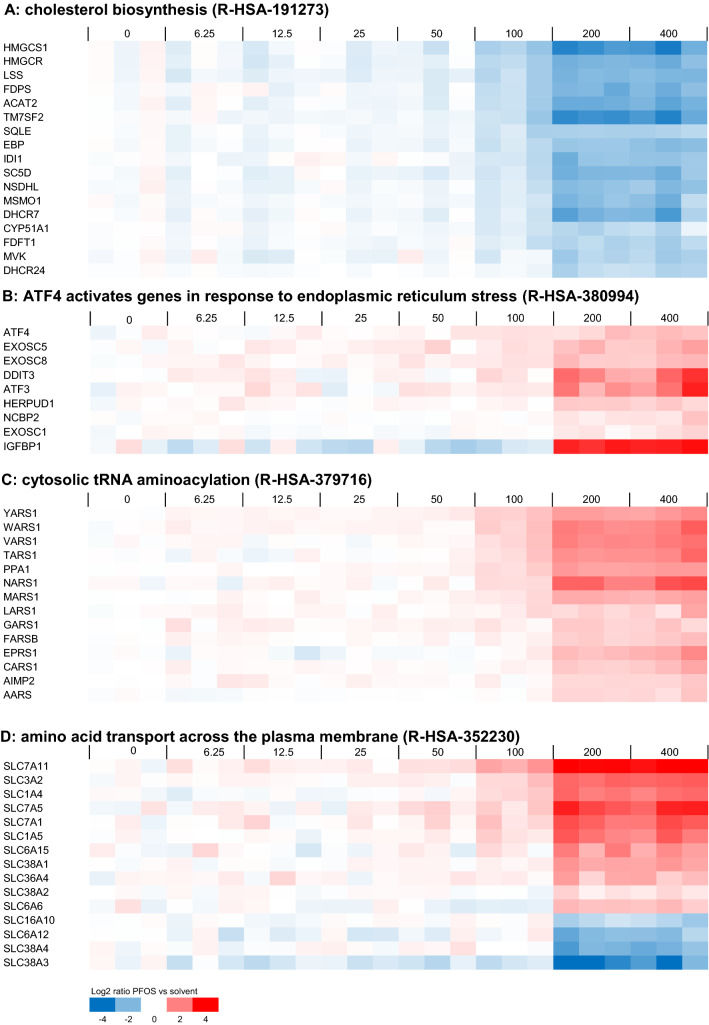
Concentration-dependent modulation of genes belonging to a selection of Reactome gene sets that are regulated in HepaRG cells upon PFOS exposure. Regulated gene sets presented here are **A** ‘cholesterol biosynthesis’ (R-HSA-191273), **B** ’ATF4 activates genes in response to endoplasmic reticulum stress’ (R-HSA-380994), **C** ‘cytosolic tRNA aminoacylation’ (R-HSA-379716), and **D** ‘amino acid transport across the plasma membrane’ (R-HSA-352230). For each PFOS exposure (concentration given in µM above the plots), data from three samples (three independent studies) are shown. Expression is normalized against average expression of the solvent control (0), showing the Log2 ratio of expression upon PFOS treatment versus expression in the control

HepaRG cells were exposed for 24 h to 0 (solvent control), 6.25, 12.5, 25, 50, 100, 200, or 400 µM PFOS and subjected to DNA microarray analysis. Data were analyzed using BMDExpress as described in the Materials and Methods section. With the applied criteria for the identification of regulated gene sets (Fisher’s Exact Two Tail ≤ 0.1, number of genes that passed all filters of a gene set ≤ 5, and the percentage of genes of the gene set regulated ≥ 20, see Materials and Methods section), 18 Reactome gene sets were upregulated (≥ 60% of the regulated genes upregulated) and 90 downregulated (≥ 60% of regulated genes downregulated). Figure 3 shows for each of the 108 regulated gene sets the percentage of genes that is affected by PFOS plotted against the median BMC value of the regulated genes. One can conclude that, in general, high micromolar concentrations of PFOS are required to cause effects and that differences in effect concentrations between gene sets are considered minor, based on the comparison of median BMC values. Gene sets related to cellular processes that were previously identified to be affected by PFOA, PFNA, and PFOS (Louisse et al. 2020) are indicated in Fig. 3. For the selection of genes to assess differences in potencies between different PFASs, genes related to these gene sets may be of particular interest, as these have been shown before to be regulated by at least three PFASs in HepaRG cells (Louisse et al. 2020). The expression data for the regulated genes for these selected gene sets are presented in Fig. 4. It must be noted that another 11 Reactome gene sets were identified to be regulated using the applied selection criteria (Fisher’s Exact Two Tail ≤ 0.1, number of genes that passed all filters of a gene set ≤ 5, and the percentage of genes regulated of the gene set ≥ 20, see Materials and Methods section) that were not clearly up- or downregulated, i.e., 40–60% of the regulated genes were upregulated and the other 40–60% of the regulated genes were downregulated. More detailed information on the results of the BMDExpress analysis for all these 119 regulated gene sets (90 downregulated, 18 upregulated, 11 not clearly up- or downregulated) is provided in the Supplementary Materials.

**Fig. 5:**
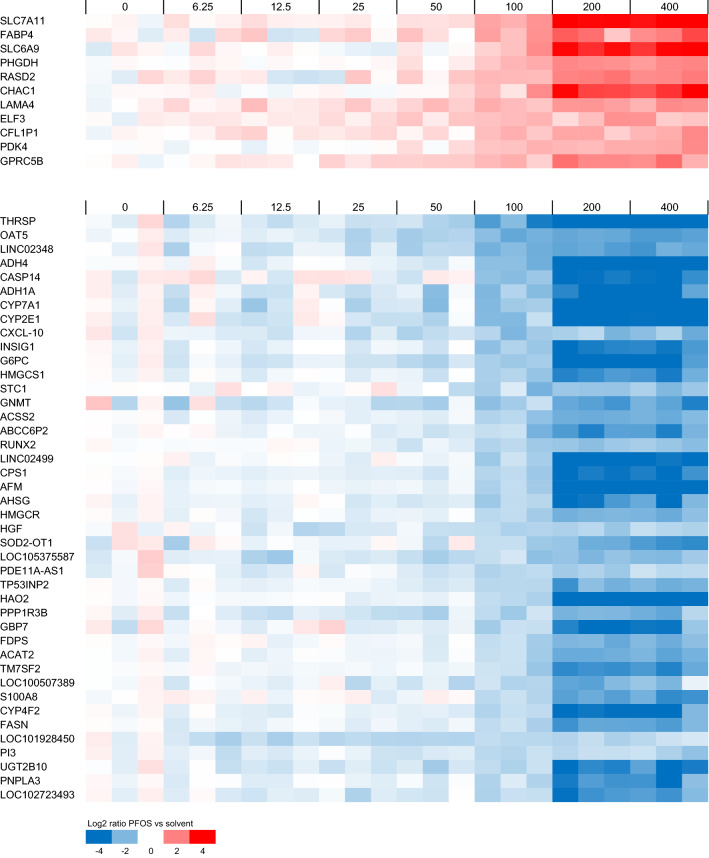
Concentration-dependent modulation of selected sensitive genes in HepaRG cells upon PFOS exposure. Data for genes are presented for which a BMC was obtained and that showed an average fold change at 100 µM of at least 2 compared to the solvent control. For each PFOS exposure (concentration given in μM above the plots), data from three samples (three independent studies) are shown. Expression is normalized against average expression of the solvent control (0), showing the Log2 ratio of expression upon PFOS treatment versus expression in the control

As a next step, the concentration–response data were analyzed to identify genes that were relatively sensitive to PFOS treatment. Besides those selected from gene sets as indicated in Figs. 3 and 4, such genes may be good candidates to assess relative potency differences between PFASs, as also PFASs with a relatively low potency may induce a response. For this, genes were selected for which a BMC value was obtained and that showed at 100 µM at least a twofold change compared to the solvent control. Microarray expression data of these genes are presented in Fig. 5. It is of interest to note that some of these are part of the selected gene sets presented in Fig. 4, whereas many are not.

**Fig. 6:**
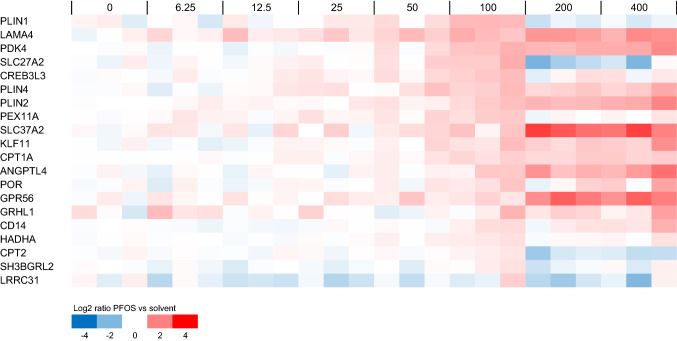
Concentration-dependent modulation of PPARα-regulated genes by PFOS in HepaRG cells. Data for genes are presented that were previously shown to be induced by the PPARα agonist GW7646 (Wigger et al. 2019) and by PFOS (Louisse et al. 2020) in HepaRG cells. For each PFOS exposure (concentration given in μM above the plots), data from three samples (three independent studies) are shown. Expression is normalized against average expression of the solvent control (0), showing the Log2 ratio of expression upon PFOS treatment versus expression in the control

In addition, the microarray data for PPARα response genes were examined, given that PPARα is a cellular target often mentioned in relation with PFAS-induced (liver) toxicity. Figure 6 shows the microarray data for PFOS-exposed cells for genes that were previously shown to be regulated by both the PPARα agonist GW7646 (Wigger et al. 2019) and by PFOS (Louisse et al. 2020) in HepaRG cells. It is of interest to note that some of these genes showed a non-typical concentration–response (*PLIN1*, *SLC27A2*, *CPT2*), i.e., showing a concentration-dependent increase in expression up to and including 100 µM, and a decrease at 200 and 400 µM (Fig. 6).

## Selection of genes for RT-qPCR analysis to assess potency differences of 18 PFASs

Subsequently, the concentration–response microarray data presented in Figs. 4, 5, 6 were analyzed in more detail to select genes suitable for analyzing the concentration-dependent effects of 18 PFASs (Fig. 1) and to provide insights into potency differences. To that end, genes were selected that showed clear concentration–response curves for PFOS and covering diverse biological processes, as well as genes with relatively low BMC values. The ten genes selected include five genes that were upregulated and five that were downregulated upon PFOS treatment (see concentration–response data for microarray data in Supplementary Fig. 6), and are shortly described below.

- *ATF4*: Activating transcription factor 4 (ATF4) is a transcription factor activated upon endoplasmic reticulum stress and/or amino acid starvation (Harding et al. 2000), upregulating genes that play a role in cell recovery, adaptation to stress conditions, and restoration of cell homeostasis (Rozpedek et al. 2016). Member of the upregulated gene set ‘ATF4 activates genes in response to endoplasmic reticulum stress’ (Fig. 4B).
- *SLC7A11*: The *SLC7A11* gene codes for an amino acid transporter importing cysteine and exporting glutamate. It is one of the amino acid transporters that is upregulated by ATF4 upon amino acid starvation (Adams 2007; Krokowski et al. 2013; Han et al. 2013; Shan et al. 2016). Member of the upregulated gene set ‘Amino acid transport across the plasma membrane’ (Fig. 4D). Highly upregulated even at relatively low PFOS concentrations (Fig. 5).
- *YARS1*: Tyrosyl-tRNA synthetase (YARS) is an aminoacyl-tRNA synthetase (ARS) catalyzing the aminoacylation of transfer RNA (tRNA) by its cognate amino acid tyrosine. It is one of the ARS genes that is upregulated by ATF4 upon amino acid starvation (Adams 2007; Krokowski et al. 2013; Han et al. 2013; Shan et al. 2016). Member of the upregulated gene set ‘Cytosolic tRNA aminoacylation’ (Fig. 4C).
- *PDK4*: pyruvate dehydrogenase (PDH) kinase 4 (PDK4) (Kwon and Harris 2004) diminishes PDH activity, thereby reducing the conversion of pyruvate to acetyl-CoA. PDK4 expression has been reported to be upregulated upon fasting and/or a switching from glucose to fatty acids as an energy source (Zhang et al. 2014; Pettersen et al. 2019). PDK4 expression has been reported to be regulated by retinoic acid receptors (Kwon and Harris 2004) and by PPARα (e.g., Wigger et al. 2019). Thus, considered to be a PPARα response gene (Fig. 6). Highly upregulated even at relatively low PFOS concentrations (Fig. 5).
- *ANGPTL4*: angiopoietin-like protein 4 (ANGPTL4) is a member of the angiopoietin-related family, and has been reported to play a crucial role in regulating angiogenesis and glucolipid metabolism (Hato et al. 2008). Regulation of *ANGPTL4* gene expression has been reported to be mediated via PPARs and HIF-1α (La Paglia et al. 2017). Thus, considered to be a PPARα response gene (Fig. 6).
- *LSS*: The protein encoded by the *LSS* gene catalyzes the conversion of (S)-2,3 oxidosqualene to lanosterol in the cholesterol biosynthesis pathway (Wada et al. 2020). Member of the downregulated gene set ‘Cholesterol biosynthesis’ (Fig. 4A).
- *HMGCR*: The gene codes for HMG-CoA reductase, the rate-limiting enzyme in the cholesterol biosynthetic pathway, which catalyzes the conversion of HMG-CoA to mevalonic acid (Luskey and Stevens 1985). Member of the downregulated gene set ‘Cholesterol biosynthesis’ (Fig. 4A). Highly downregulated even at relatively low PFOS concentrations (Fig. 5).
- *OAT5*: Organic anion transporter 5 (OAT5) is an anion exchanger. Expression in the liver has been reported to be regulated via hepatocyte nuclear factor-1α (HNF-1α) (Klein et al. 2010). Highly downregulated even at relatively low PFOS concentrations (Fig. 5).
- *THRSP*: Thyroid hormone responsive (THRSP) is primarily a nuclear protein that plays a role in the regulation of lipid metabolism. Expression has been reported to be downregulated upon fasting (Kuemmerle and Kinlaw 2011). Highly downregulated even at relatively low PFOS concentrations (Fig. 5).
- *CXCL10*: C-X-C motif chemokine ligand 10 (CXCL10) is a chemokine capable of stimulation of monocytes, natural killer cell and T cell migration, regulation of T cell and bone marrow progenitor maturation, modulation of adhesion molecule expression, and inhibition of angiogenesis (Neville et al. 1997). Highly downregulated even at relatively low PFOS concentrations (Fig. 5).

## Effects of 18 PFASs on expression of selected genes

**Fig. 7:**
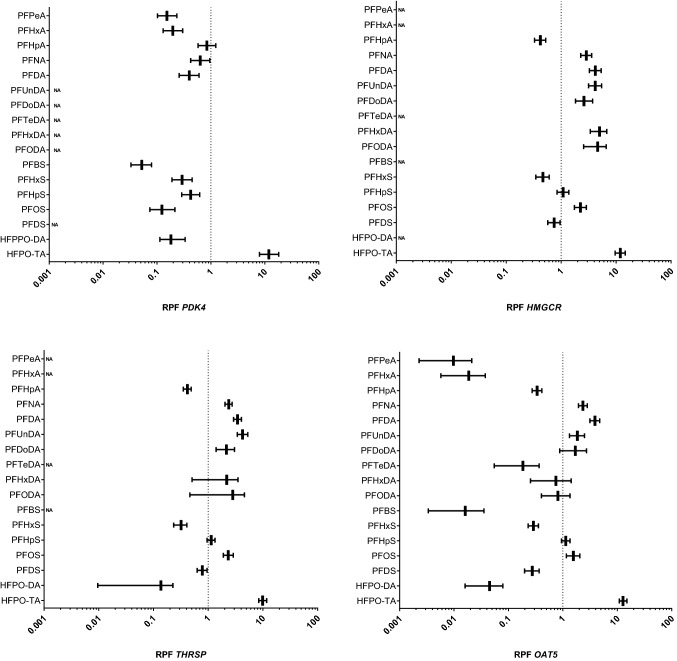
In vitro RPFs based on PROAST dose–response analysis of *PDK4*, *HMGCR*, *OAT5*, and *THRSP* gene expression data obtained from HepaRG cells exposed to various PFASs. RPFs are presented as vertical lines, with the 5% lower bound and 95% upper bound of the confidence interval as whiskers. PFOA was used as index chemical, i.e., has an RPF of 1 (dotted line). *NA* not applicable, RPF could not be determined

To determine the relative potencies of the 18 PFASs, the HepaRG cell line was exposed for 24 h to increasing concentrations of the 18 PFASs shown in Fig. 1. After exposure, RNA was collected and used for RT-qPCR analysis of the ten selected genes. Supplementary Fig. 7 shows concentration–response data of these genes for PFOS, PFOA and HPFO-TA, the latter being the PFAS that was found to be most potent in the present study based on cell viability and triglyceride accumulation as well as for gene expression modulation. Concentration–response data for the 18 PFASs for all genes are presented in the Supplementary Materials. These data were then used to perform PROAST dose–response analysis using parallel curve fitting to obtain in vitro RPFs related to PFAS-induced gene expression changes. For the selected genes, only for *OAT5* RPFs could be obtained for all tested PFASs (18 including PFOA). For *CXCL10* and *THRSP*, RPFs were obtained for 14 PFASs, for *LSS*, *HMGCR* and *ANGPTL4* for 13 PFASs, for *ATF4* and *PDK4* for 12 PFASs, and for *SLC7A11* and *YARS1* for 11 PFASs. Figure 7 presents the RPFs based on gene expression data for *PDK4*, * HMGCR*, *OAT5*, and *THRSP*. RPFs for all genes are presented in Supplementary Fig. 8.

Gene-specific differences in RPF values were observed, although some general patterns could be identified. In general, RPFs obtained for the PPAR response genes *PDK4* and *ANGPTL4* were similar, but differed for many PFASs from RPFs obtained from the other genes (Supplementary Figs. 8 and 9). For *PDK4* and *ANGPTL4*, all studied PFASs, except HFPO-TA, were less potent than PFOA. For the majority of the other genes (*ATF4*, *SLC7A11*, *YARS1*, *LSS*, *HMGCR*, *OAT5*, and *THRSP*), PFNA, PFDA, PFUnDA, PFDoDA, PFHpS, PFOS, and HFPO-TA were consistently more potent than PFOA, and PFHpA, PFHxS, and PFDS less potent than PFOA.

**Fig. 8:**
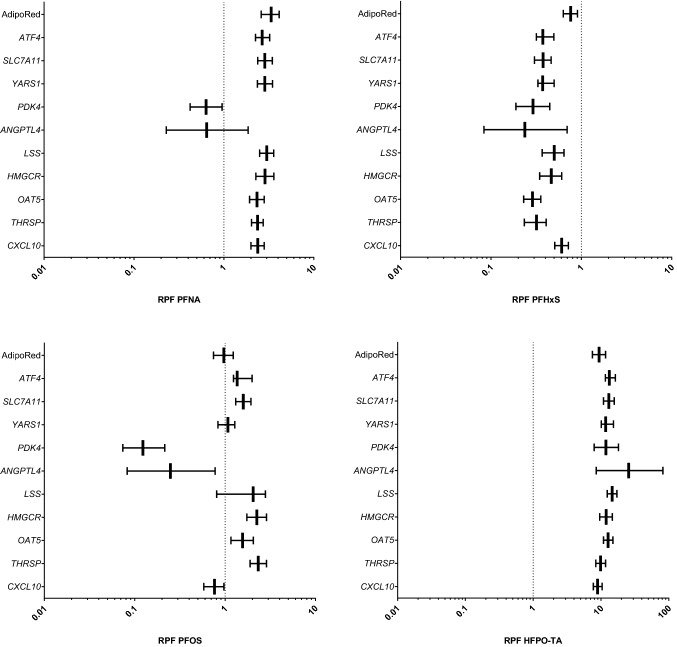
In vitro RPFs based on PROAST dose–response analysis of gene expression and AdipoRed data obtained from HepaRG cells exposed to various PFASs. RPFs are presented as vertical lines, with the 5% lower bound and 95% upper bound of the confidence interval as whiskers. PFOA was used as index chemical, i.e., has an RPF of 1 (dotted line)

For PFNA, PFHxS, PFOS, and HFPO-TA in vitro RPFs were obtained for all readouts (AdipoRed data and gene expression data) (Fig. 8), whereas for other PFASs, this was not the case (Supplementary Fig. 9). Of these 4 PFASs, RPFs related to all in vitro readouts were smaller than 1 for PFHxS. RPF patterns of PFPeA, PFHxA, PFHpA, PFBS, PFDS, and HFPO-DA were similar as for PFHxS, i.e., having in general RPFs lower than 1 (Supplementary Fig. 9). HFPO-TA was the only PFAS tested for which all in vitro RPFs were found to be larger than 1. For PFNA, RPFs related to expression of PPAR response genes (*PDK4* and *ANGPTL4*) were smaller than 1, whereas these were larger than 1 for the other readouts. For PFOS, potencies for the two PPAR response genes and *CXCL10* were lower than those of PFOA, whereas for other genes, these were similar or slightly higher. PFHpS showed a similar RPF pattern as that of PFOS, as well as PFDoDA, although for the latter PFAS no RPFs could be determined for the PPAR response genes (Supplementary Fig. 8). For the longer-chain PFASs PFTeDA, PFHxDA, and PFODA, RPFs were only obtained for 2, 4, and 5 readouts, respectively (Supplementary Fig. 9).

**Fig. 9:**
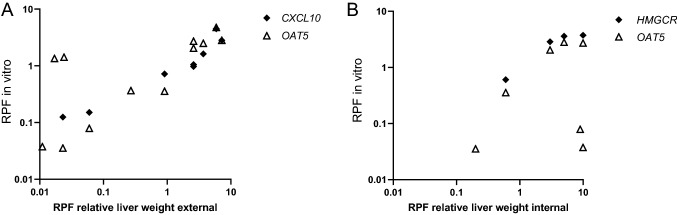
Comparison of **A** in vitro RPFs based on *OAT5* or *CXCL10* gene expression data with reported external RPFs for PFAS-induced liver toxicity in rats and **B** in vitro RPFs based on *OAT5* or *HMGCR* gene expression data with reported internal RPFs for PFAS-induced liver toxicity in rats

We then performed a Spearman correlation analysis using GraphPad Prism 9 to assess whether potency rankings obtained with different in vitro readouts are correlated and to assess whether certain in vitro-based potency rankings correlate with in vivo potency rankings based on reported external in vivo RPFs (Bil et al. 2021, 2022a) or internal in vivo RPFs (Bil et al. 2022b). The results of the correlation analysis point to a reasonable correlation between most of the in vitro RPFs, except for *ANGPTL4* and *PDK4*, both PPAR target genes (Supplementary Fig. 10). A reasonable correlation was found between the in vitro RPFs based on *CXCL* or *OAT5* expression and external in vivo RPFs (Supplementary Fig. 10). Regarding internal RPFs, the best correlation was found for *HMGCR* expression, but it must be noted that this was only based on data for 4 PFASs. Figure 9 presents the external and internal in vivo RPFs in comparison with the in vitro RPFs for *OAT5* and *CXCL10* expression, and *OAT5* and *HMGCR* expression, respectively. Although in vitro RPFs based on changes in *OAT5* expression correlate well with external in vivo RPFs (Supplementary Fig. 10), PFHxDA and PFODA are major outliers, showing in vitro RPFs > 1 and in vivo external RPFs < 0.1 (Fig. 9A). The slightly better correlation between in vitro RPFs based on *CXCL10* expression and external in vivo RPFs (Supplementary Fig. 10), may relate to the fact that for PDHxDA and PFODA, no in vitro RPFs could be obtained (Supplementary Figs. 8 and 9), being therefore excluded from the correlation analysis. The in vitro RPFs correlate to a lesser extent to internal RPFs than to external RPFs (Supplementary Fig. 10), showing the best correlation for RPFs based on *HMGCR* expression (Fig. 9B). As indicated above, this was only based on data for 4 PFASs. When comparing in vitro RPFs based on *OAT5* expression with internal in vivo RPFs, it becomes clear that PFHxA and HFPO-DA are the main outliers, showing in vitro RPFs < 0.1 and in vivo RPFs > 1 (Fig. 9B). All in vitro and in vivo RPFs used for these analyses are presented in an Excel file that can be found in the Supplementary Materials.

## Discussion

The present study evaluated the in vitro toxicity of 18 PFASs in human HepaRG liver cells, by studying the effects on cellular triglyceride accumulation and gene expression changes, and assessed whether these in vitro data can be used to obtain insight into potency differences regarding hepatotoxicity of PFASs. In vitro RPFs could be obtained for 8 PFASs (including index chemical PFOA) based on the triglyceride accumulation data, whereas for the selected genes in vitro RPFs could be obtained for 11–18 PFASs (including index chemical PFOA). Only for PFNA, PFHxS, PFOS, and HFPO-TA in vitro RPFs were obtained for all readouts. For the readout *OAT5* expression, in vitro RPFs were obtained for all PFASs. In vitro RPFs were found to correlate in general well with each other (Spearman correlation) except for the PPAR target genes *ANGPTL4* and *PDK4*. Comparison of in vitro RPFs with reported in vivo RPFs in rats indicate that best correlations (Spearman correlation) were obtained for in vitro RPFs based on *OAT5* and *CXCL10* expression changes and external in vivo RPFs. HFPO-TA was found to be the most potent PFAS tested, being around tenfold more potent than PFOA.

To assess effects of PFASs on triglyceride accumulation, we applied the AdipoRed assay. Interestingly, we found for the PFASs more pronounced effects (and better concentration-dependent effects) upon a 24-h exposure than upon a 72-h exposure, in contrast to the fungicide cyproconazole, for which a 72-h exposure was found to show most effects, and which was used as a model steatotic compound in an in vitro study on adverse outcome pathway (AOP)-driven analysis of liver steatosis (Luckert et al. 2018). This may relate to different modes of action underlying chemical-induced steatotic effects, as indicated by the available AOPs on this endpoint (Vinken 2013, 2015; Mellor et al. 2016). It is of interest to note that upon a 72-h exposure, the AdipoRed signal returned in various exposure conditions for PFHxS and PFOS to the same levels as in the solvent control (Supplementary Fig. 4). The toxicological meaning of that finding is not clear, but it may point to a possible cellular response to increased cellular triglyceride levels at earlier time points. Various studies have shown a PFAS-induced increase of hepatic triglyceride levels in experimental animals. PFOA, PFNA, PFHxS and PFOS have been shown to increase hepatic triglycerides in male mice (Bijland et al. 2011; Das et al. 2017; Huck et al. 2018; Hui et al. 2017; Wan et al. 2012). As indicated in recent Opinions of the EFSA CONTAM Panel, thorough knowledge of the mode of action underlying the development of hepatocellular steatosis in PFAS-treated rodents is missing (EFSA CONTAM Panel 2018, 2020).

Although we identified some genes that can be considered relevant readouts to screen PFASs for possible liver toxicity, one would like to mechanistically relate the gene expression change(s) to adverse effects to the liver. Ideally such gene expression changes would be a key event (KE) of an AOP related to liver toxicity. The AOP-wiki was consulted to assess whether in vitro effects measured in the present study are part of (putative) AOPs related to liver toxicity (https://aopwiki.org/; latest access: 28-12-2022). Of the in vitro readouts of the present study, triglyceride accumulation was found in the AOP-wiki as proposed key event related to liver steatosis. In light of the possible endoplasmic reticulum stress induced by the PFASs tested (indicated by activation of ATF4 signaling), it is of interest to note that the updated AOP on liver steatosis (from Mellor et al. (2016) based on earlier work of Vinken (2013; 2015)) includes an induction of endoplasmic reticulum stress as a key event following increase of triglyceride accumulation. The selected genes are not present as key events in the AOPs present in the AOP-wiki, but it may still be possible that changes in expression of the genes can be related to certain KEs of relevance for liver toxicity, which would require a more extensive assessment. In the Supplementary Materials, some more information on the possible link of gene expression changes of the selected genes assessed in the present study in relation to (liver) toxicity is provided.

For comparison of the potencies of the various PFASs on the basis of transcriptomics data, different approaches can be followed. For example, benchmark concentration gene accumulation plots may be used for potency ranking (Ramaiahgari et al. 2019; Reardon et al. 2021). Furthermore, transcriptomics (TempO-Seq) data obtained upon exposure of human primary liver cell spheroids to a large number of different PFASs have been analyzed applying BMDExpress and potencies were derived and compared using the median benchmark concentration of all filtered genes that adhere to best-fit models (Rowan-Caroll et al. 2021; Reardon et al. 2021). Although these are meaningful approaches, we have chosen to determine transcriptional benchmark concentrations for individual differentially expressed genes using PROAST since this BMD modeling software allows for parallel curve fitting and has recently been used for the calculation of in vivo RPFs of various PFASs (Bil et al. 2021, 2022a, b). When comparing RPFs obtained for the different readouts, it was shown that for most readouts good correlations were found. However, correlations were rather poor for the PPAR response genes *PDK4* or *ANGPTL4* and the other readouts. It must be noted that for the 8 genes for which the RPFs correlate well, still considerable differences in RPFs exist. It is difficult to select one gene that would provide the best data on relative potencies, and it can be expected that the study set-up, including the choice of exposure time (24 h in the present study) will affect the RPFs obtained. The data should therefore rather be used to obtain a general indication of whether a certain PFAS is expected to be a relatively potent hepato-toxicant or whether it will be of less concern related to its hepatotoxic effects. As we obtained RPFs for all PFASs based on changes in *OAT5* expression, the comparison of *OAT5*-based RPFs with available external and internal RPFs reported in the literature is of specific interest (Fig. 9). From that comparison, in vitro RPFs were in general good in line with RPFs based on in vivo studies, with most striking exceptions for PFHxDA and PFODA for external RPFs and PFHxA and HFPO-DA for internal RPFs. The discrepancy for PFHxDA and PFODA regarding external RPFs (high in vitro RPFs vs low external in vivo RPFs) may relate to a relatively low systemic uptake of these large molecules upon oral exposure. Relative differences in systemic exposure are accounted for using internal RPFs, for which kinetic models were applied to estimate internal exposure (Bil et al. 2022b). Internal RPFs are, however, not available for PFHxDA and PFODA. The discrepancy for PFHxA and HFPO-DA regarding internal RPFs (low in vitro RPFs vs high internal in vivo RPFs) is more difficult to explain. It is of interest to further investigate these in vitro–in vivo differences in future studies. They may, among others, relate to possible species differences in PFAS-induced effects on the liver (Fragki et al. 2021). Of course, also differences in exposure duration or other differences between the in vitro and in vivo situation may play a role. Studies that assess possible species differences in human and rat liver cells in vitro may shed more light on this. It shall be noted here that the evaluation of the predictive capability of in vitro assays should not necessarily be based on a comparison to animal in vivo data (van der Zalm et al. 2022). Ideally, one would like to compare the in vitro HepaRG data with effect data in humans. Epidemiological evidence has correlated PFOS and PFOA exposure to a small elevation in serum levels of the hepatic enzyme ALT (alanine transferase), a biomarker for liver damage (Gallo et al. 2012). As indicated before, it is questionable whether that limited increase in ALT is causally related to PFOS and PFOA exposure, and whether it reflects serious liver damage. Also, data on other PFASs are scarce or lacking, making these in vitro human vs in vivo human comparisons cumbersome. To obtain in vivo relative potencies based on in vitro toxicity data, information on toxicokinetics should be included in the assessment. In that regard, we have been working on the quantitative in vitro to in vivo extrapolation (QIVIVE) of the toxicity data of PFOA, PFNA, PFHxS, and PFOS, translating cell-associated PFAS levels to oral equivalent doses using physiologically based kinetic (PBK) modeling, providing information that will be of use in the assessment of relative potencies of PFASs in humans (Fragki et al. 2023).

Although the main aim of this study was to select in vitro readouts related to liver toxicity that can be used to determine in vitro potency differences for PFASs, the obtained concentration–response microarray data may be of use to increase our insights into mechanisms related to the liver toxicity of PFASs in humans. The BMDExpress analysis indicated 18 gene sets to be upregulated and 90 gene sets to be downregulated. Many of the regulated gene sets are related to cholesterol biosynthesis and lipid metabolism as also indicated by Rowan-Carroll et al. (2021), who assessed the concentration- and time-dependent effects of PFOA, PFBS, PFOS and PFDS on gene expression in human primary hepatocyte spheroids. In a later study, this work was extended to include more PFASs and to estimate relative potencies (Reardon et al. 2021), testing carboxylates (PCFAs), sulfonates (PFSAs) and fluorotelomers and sulfonamides. In general, PFCAs and PFSAs caused gene expression changes with increased potency with increasing carbon chain-length (Reardon et al. 2021), being in line with findings for some of the genes in the present study. In general, effective concentrations in the present study are for most genes in the high micromolar range, which are not expected to be reached in vivo in relevant exposure scenarios. Rowan-Carroll et al. (2021) and Reardon et al. (2021) found effects at low micromolar concentrations, which may relate to the difference in test system used (2D culture HepaRG cells in present study vs. 3D primary hepatocyte model) as well as difference in exposure duration (24 h in the present study vs. up to 14 days in the study of Rowan-Carroll et al. (2021) and up to 10 days in the study of Reardon et al. (2021)). We recently showed that HepaRG cells cultured in an organ-on-a-chip device can be cultured for at least 8 weeks, allowing chronic exposure studies (Duivenvoorde et al. 2021). Such long-term studies may provide more insights into effects at more relevant human effect concentrations, but given the low throughput, such models are less suitable for screening a large number of PFASs.

Of the PFASs tested in the present study, HFPO-TA was shown to be the most potent. Sheng et al. (2018) assessed the effects of HPFO-TA in mice and concluded it to be a potent hepatotoxicant, causing hepatomegaly, necrosis, and increase in serum ALT, as well as a dose-dependent decrease in total cholesterol and triglycerides in the liver, and they concluded it to be more potent than PFOA, which was tested in an earlier study from the same group (Yan et al. 2014). In 2017, Pan and coworkers were the first to report on the environmental occurrence (Xiaoqing River in China), bioaccumulation (in carp) and presence in human serum of HFPO-TA, concluding that the emerging usage of HFPO-TA in the fluoropolymer manufacturing industry raises concerns about its toxicity and potential health risks to aquatic organisms and humans (Pan et al. 2017). In a more recent study, HFPO-TA was measured in the serum of residents living near a fluorochemical plant in Shandong, China, showing median serum concentrations of ~ 2 ng/mL (low pM range), almost 100 times lower than the median PFOA serum concentrations of these individuals (Yao et al. 2020). Based on our in vitro studies, which seems to be in line with the limited in vivo evidence (Sheng et al. 2018), HFPO-TA is a rather toxic PFAS, suggesting that its production and/or application should be discouraged and that human exposure should be prevented.

Altogether, the present study shows an approach to select in vitro gene expression readouts in HepaRG cells that can be used to obtain information on relative potencies of PFASs related to liver toxicity in vitro. It may be concluded that the HepaRG model may provide relevant data to get insight into which PFASs are relevant regarding their hepatotoxic effects and that it can be applied as a screening tool to prioritize other PFASs for further hazard and risk assessment.

## Supplementary Information

Below is the link to the electronic supplementary material.

Supplementary file1 (XLSX 209 KB)

Supplementary file2 (TXT 5272 KB)

Supplementary file3 (XLSX 95 KB)

Supplementary file4 (CSV 8579 KB)

Supplementary file5 (TXT 308 KB)

Supplementary file6 (XLSX 18 KB)

Supplementary file7 (DOCX 971 KB)

## Data Availability

The datasets generated and analyzed during the current study can either be found in the supplementary information or are available from the corresponding author on reasonable request.
