# Supplementary material for: Determination of in vitro hepatotoxic potencies of a series of perfluoroalkyl substances (PFASs) based on gene expression changes in HepaRG liver cells
Source: Arch Toxicol. 2023 Mar 3;97(4):1113–31. doi: 10.1007/s00204-023-03450-2 (PMC10025204; doi:10.1007/s00204-023-03450-2)
Supplement: Supplementary file 7 — Supplementary file7 (DOCX 971 KB) [file 204_2023_3450_MOESM7_ESM.docx]

*Supplementary Table 1. Suppliers, purity, catalog numbers, CAS numbers and maximum concentrations of PFASs tested in the present study.*

| PFAS | Full name | Supplier | Purity | Catalog number | CAS number | Highest tested concentration (µM) |
| --- | --- | --- | --- | --- | --- | --- |
| PFPeA | perfluoropentanoic acid | Sigma-Aldrich | 97% | 396575-5ML | 2706-90-3 | 400 |
| PFHxA | perfluorohexanoic acid | Synquest laboratories | 97% | 2121-3-39 | 307-24-4 | 400 |
| PFHpA | perfluoroheptanoic acid | Sigma-Aldrich | 99% | 342041-5G | 375-85-9 | 400 |
| PFOA | perfluorooctanoic acid | Sigma-Aldrich | 95% | 171468-5G | 335-67-1 | 400 |
| PFNA | perfluorononanoic acid | Sigma-Aldrich | 99% | 91977-50MG | 375-95-1 | 400 |
| PFDA | perfluorodecanoic acid | Sigma-Aldrich | 98% | 177741-5G | 335-76-2 | 400 |
| PFUnDA | perfluoroundecanoic acid | Sigma-Aldrich | 95% | 446777-5G | 2058-94-8 | 400 |
| PFDoDA | perfluorododecanoic acid | Sigma-Aldrich | 95% | 406449-1G | 307-55-1 | 100 |
| PFTeDA | perfluorotetradecanoic acid | Sigma-Aldrich | 96% | 446785-5G | 376-06-7 | 100 |
| PFHxDA | perfluorohexadecanoic acid | abcr GmbH | 95% | AB 108458 | 67905-19-5 | 25 |
| PFODA | perfluorooctadecanoic acid | abcr GmbH | 95% | AB 108482 | 16517-11-6 | 25 |
| PFBS | perfluorobutane sulfonate | Sigma-Aldrich | 97% | 562629-5G | 375-73-5 | 400 |
| PFHxS | perfluorohexane sulfonate | Synquest laboratories | 95% | 6164-3-2T | 355-46-4 | 400 |
| PFHpS | perfluoroheptane sulfonate | Synquest laboratories | 99% | 6164-3-2S | 375-92-8 | 400 |
| PFOS | perfluorooctane sulfonate | Synquest laboratories | 97% | 6164-3-08 | 1763-23-1 | 400 |
| PFDS | perfluorodecane sulfonate | Toronto Research Chemicals | * | P286540 | 335-77-3 | 200 |
| HFPO-DA (GenX) | hexafluoropropylene oxide dimer acid | Synquest laboratories | 97% | 2121-3-13 | 13252-13-6 | 400 |
| HFPO-TA | hexafluoropropylene oxide trimer acid | abcr GmbH | 97% | 164194-5G | 13252-14-7 | 400 |

* Information on purity not provided by supplier.

#### Supplementary Table 2. MRM transitions of the PFASs tested for stability in the present study.

| **Name** | **Q1 Mass Da** | **Q3 Mass Da** | **Dwell (msec)** | **EP** | **CE** | **CXP** |
| --- | --- | --- | --- | --- | --- | --- |
| ^13^C_3_-HFPO-DA | 287.0 | 169.0 | 10.7 | -10 | -17 | -5 |
| HFPO-DA | 285.0 | 169.0 | 10.7 | -10 | -11 | -9 |
| HFPO-DA | 285.0 | 185.0 | 10.7 | -10 | -23 | -11 |
| HFPO-TA | 495.0 | 185.0 | 9.4 | -10 | -20 | -9 |
| HFPO-TA | 185.0 | 119.0 | 9.4 | -10 | -20 | -6 |

*
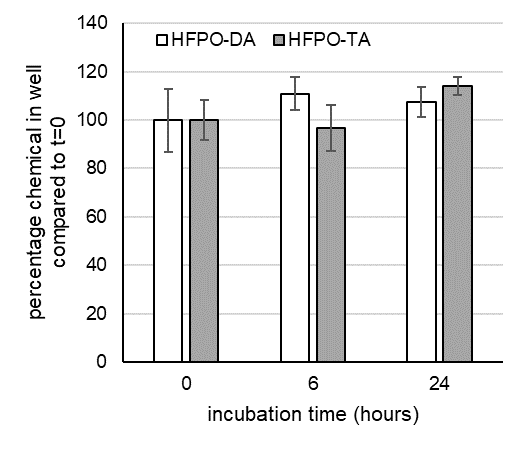
*

*Supplementary Figure 1. HFPO-DA and HFPO-TA levels in culture medium upon 0, 6 or 24 hour incubation.*


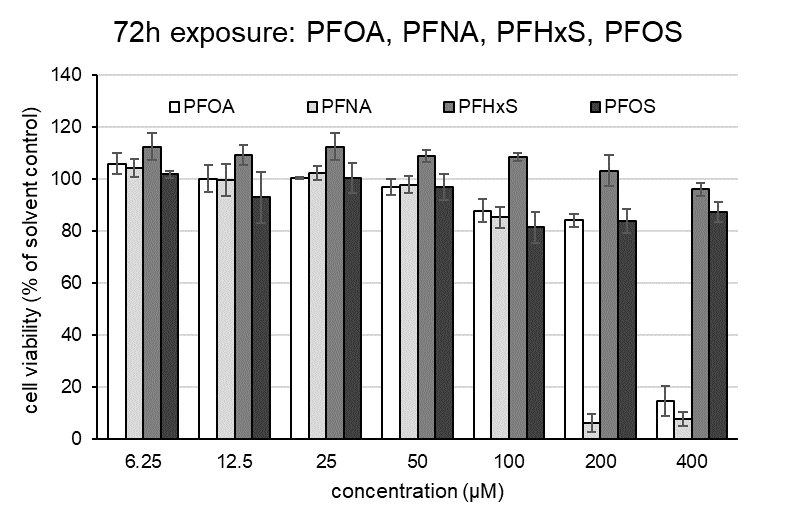


*Supplementary Figure 2. Effect of 72-h exposure to PFOA, PFNA, PFHxS or PFOS on viability of HepaRG cells as determined with the WST-1 assay and expressed as percentage of the solvent control (0.5% DMSO). Data are presented as mean ± SD of three independent experiments (using per independent experiment the mean of three technical replicates).*

*
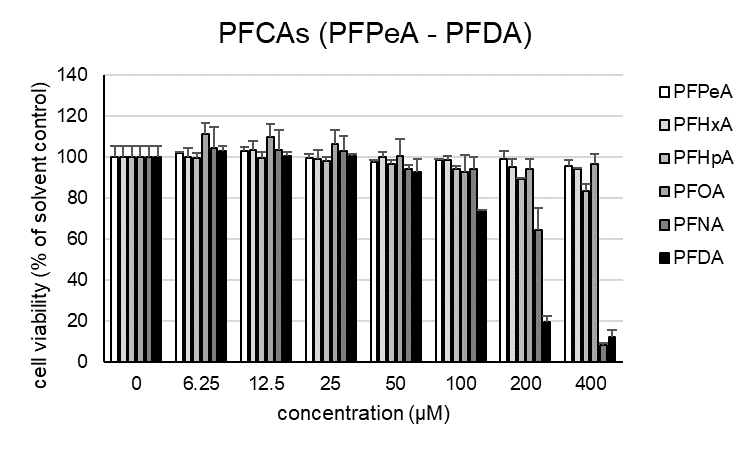

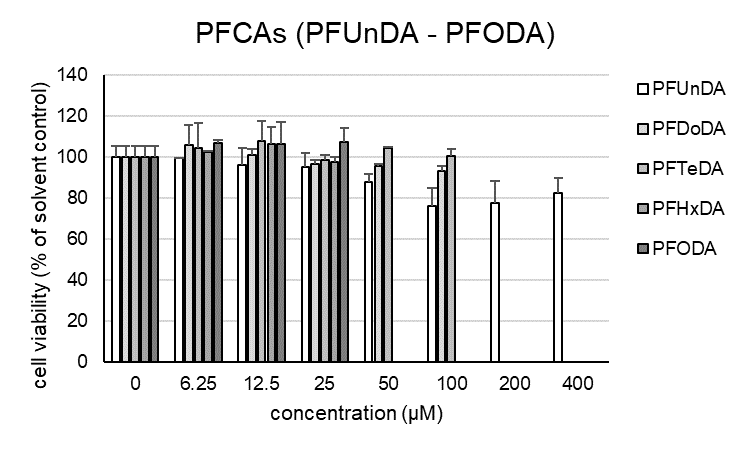

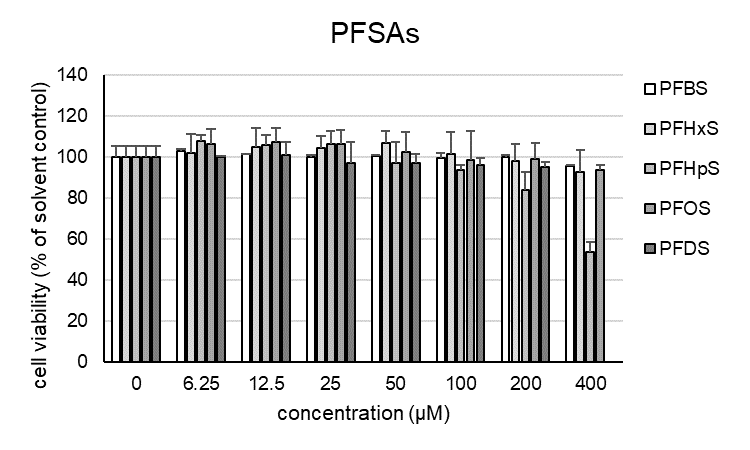
*

*
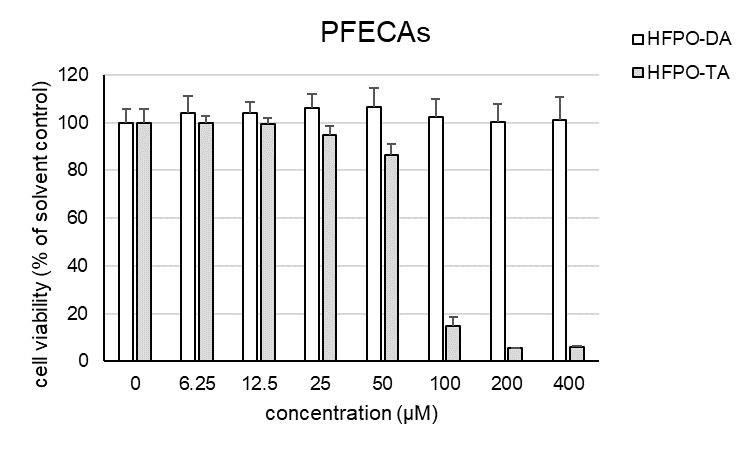
*

*Supplementary Figure 3. Effect of 24-h exposure to PFASs on viability of HepaRG cells as determined with the WST-1 assay and expressed as percentage of the solvent control (0.5% DMSO). Data are presented as mean ± SD of three independent experiments (using per independent experiment the mean of three technical replicates).*

*
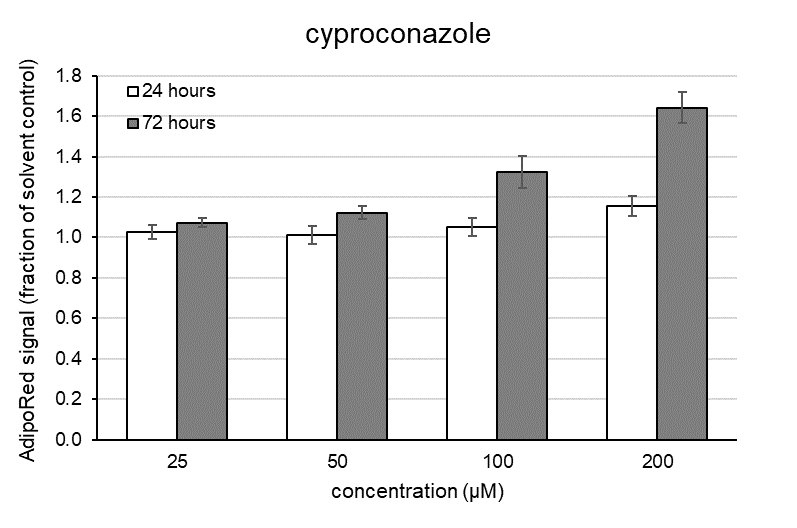

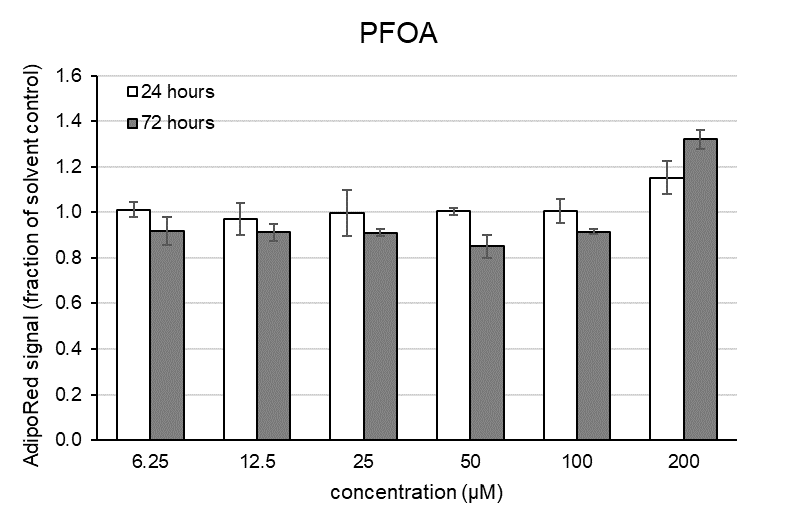

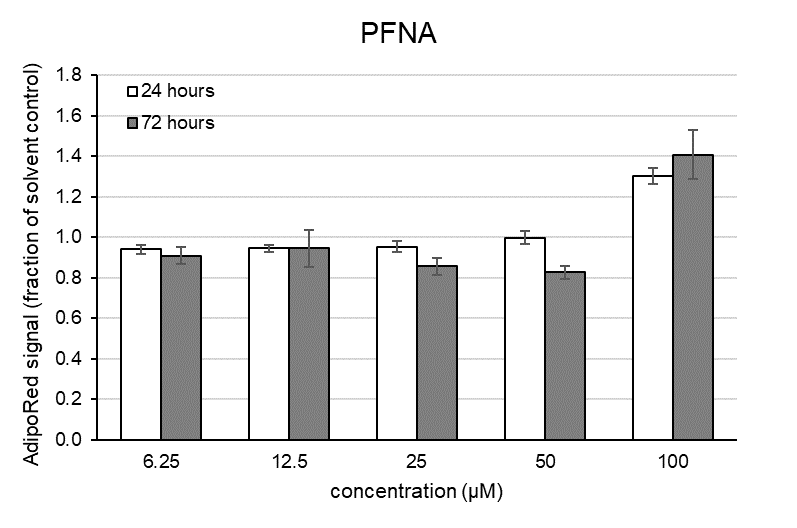

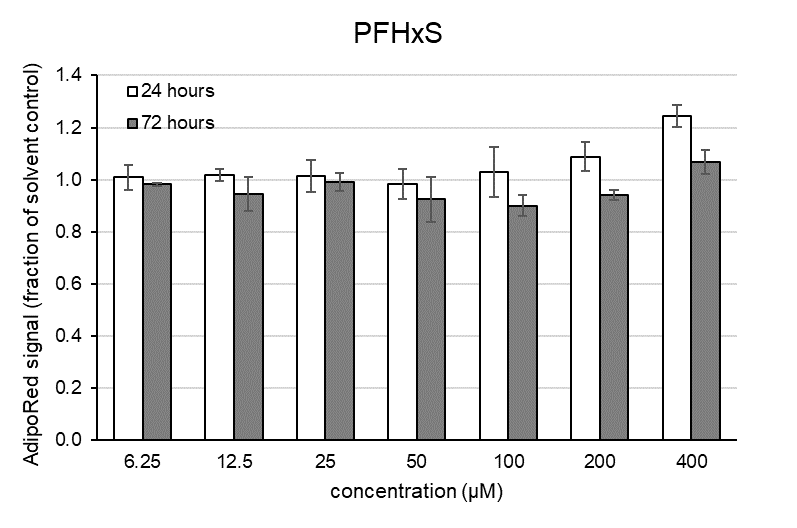

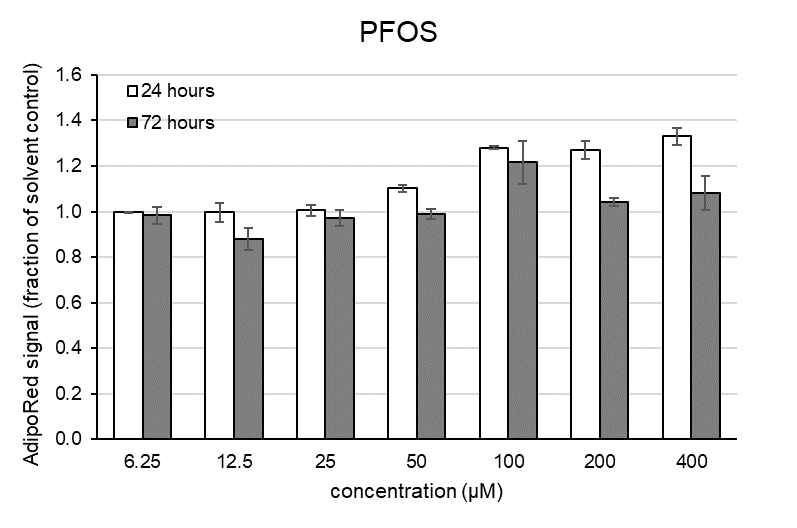
*

*Supplementary Figure 4. Effect of 24-h or 72-h exposure to cyproconazole, PFOA, PFNA, PFHxS or PFOS on triglyceride levels in HepaRG cells as determined with the AdipoRed assay and expressed as percentage of the solvent control (0.5% DMSO). Data are presented as mean ± SD of three independent experiments (using per independent experiment the mean of three technical replicates).*

*
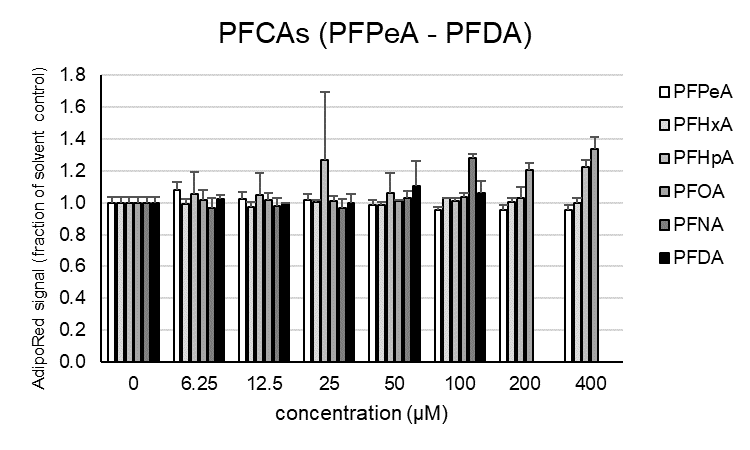

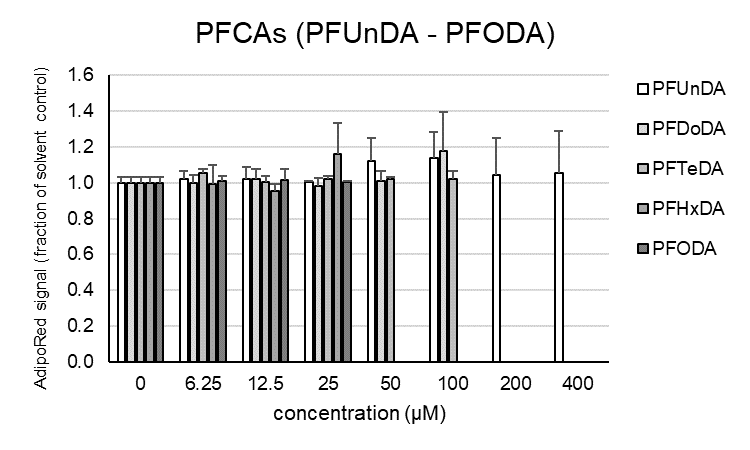

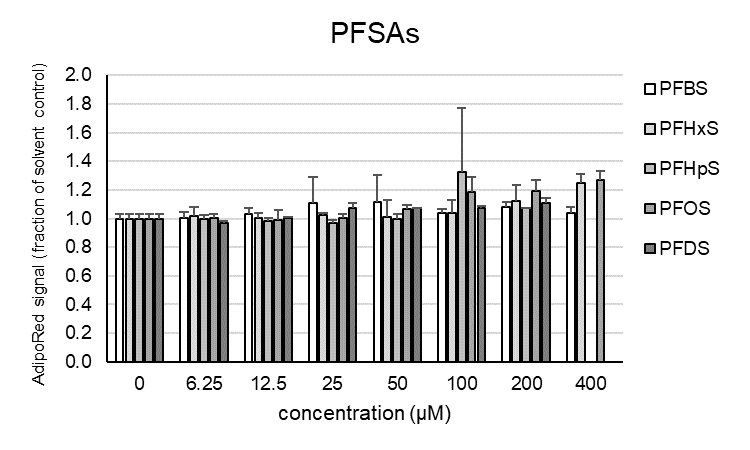
*

*
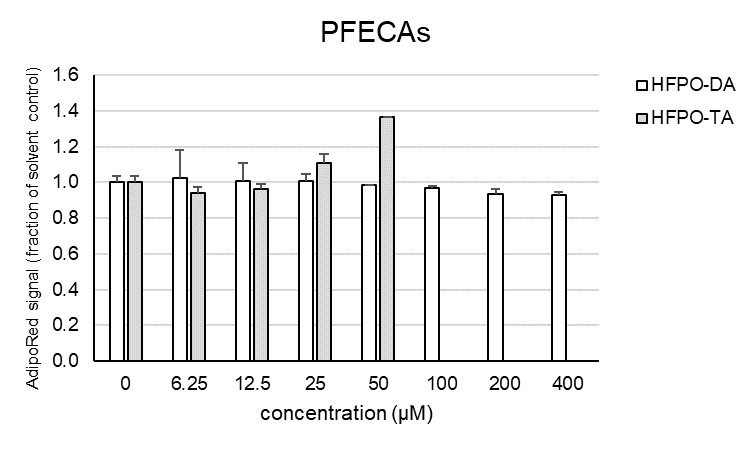
*

*Supplementary Figure 5. Effect of 24-h exposure to PFASs on triglyceride levels in HepaRG cells as determined with the AdipoRed assay and expressed as percentage of the solvent control (0.5% DMSO). Data are presented as mean ± SD of three independent experiments (using per independent experiment the mean of three technical replicates).*

***CXCL10***

concentration (µM)

concentration (µM)

concentration (µM)

concentration (µM)

concentration (µM)

concentration (µM)

concentration (µM)

concentration (µM)

concentration (µM)

concentration (µM)

gene expression (Log2 fold change compared to control)

***THRSP***

gene expression (Log2 fold change compared to control)

gene expression (Log2 fold change compared to control)

***OAT5***

***HMGCR***

***LSS***

***ANGPTL4***

***PDK4***

***YARS1***

***SLC7A11***

***ATF4***

gene expression (Log2 fold change compared to control)

*Supplementary Figure 6. Concentration-response microarray data of PFOS for selected genes to determine in vitro potency differences of PFASs. Average values and standard deviations of data from three independent studies are presented.*

concentration (µM)

concentration (µM)

concentration (µM)

concentration (µM)

concentration (µM)

***CXCL10***

concentration (µM)

concentration (µM)

concentration (µM)

concentration (µM)

concentration (µM)

gene expression (Log2 fold change compared to control)

***THRSP***

gene expression (Log2 fold change compared to control)

gene expression (Log2 fold change compared to control)

***OAT5***

***HMGCR***

***LSS***

***ANGPTL4***

***PDK4***

***YARS1***

***SLC7A11***

***ATF4***

gene expression (Log2 fold change compared to control)

*Supplementary Figure 7. Concentration-response data of PFOA- (black triangles), PFOS- (white squares) and HFPO-TA- (grey circles) induced effects on the expression of selected genes based on RT-qPCR analysis. Average values of data from three independent studies are presented.*

*Supplementary Figure 8. In vitro RPFs based on PROAST dose-response analysis of ATF4, SLC7A11, YARS1. PDK4, ANGPTL4, LSS, HMGCR, OAT5, THRSP and CXCL10 gene expression data obtained from HepaRG cells exposed to various PFASs. RPFs are presented as vertical lines, with the 5% lower bound and 95% upper bound of the confidence interval as whiskers. PFOA was used as index chemical, i.e., has an RPF of 1 (dotted line). NA: not applicable, RPF could not be determined.*

*Supplementary Figure 9. In vitro RPFs based on PROAST dose-response analysis of gene expression and AdipoRed data obtained from HepaRG cells exposed to various PFASs. RPFs are presented as vertical lines, with the 5% lower bound and 95% upper bound of the confidence interval as whiskers. PFOA was used as index chemical, i.e., has an RPF of 1 (dotted line). NA: not applicable, RPF could not be determined.*

**

B

A

**

*Supplementary Figure 10. Results of Spearman correlation analysis performed using Graphpad Prism 9, presenting obtained r-values (A) and p-values (B). Correlation with both an r-value > 0.8 and a p-value < 0.05 are accentuated by a black border.*

**Information on selected genes and possible relation to liver toxicity.**

***OAT5***

Limited information on the function and regulation of expression of OAT5 is available. Klein et al. (2010) showed that OAT5 expression in the liver is regulated via HNF-1α. A downregulation of OAT5 may therefore relate to a downregulation and/or diminished action of HNF-1α. Interestingly, HNF-1α was shown to be an essential regulator of bile acid and plasma cholesterol metabolism in mice (Shih et al., 2001). An important role of HNF-4α deregulation in PFAS-induced disturbance of lipid homeostasis has been suggested (recently reviewed by Fragki et al., 2021). Diminished HNF-1α activity may be linked to diminished HNF-4α activity, as HNF-4α has been shown to act as an HNF-1α coactivator (Eeckhoute et al., 2014). This may indicate that PFAS-induced OAT5 downregulation is possibly (also) accomplished through PFAS-induced diminished HNF-4α activity, but more focused studies would be required to conclude on this, which was out of the scope of the present study. It is of interest to mention that HNF-4α is a transcriptional activator of the rate-limiting enzyme CYP7A1 in the bile acid biosynthesis (Miao et al. 2006). Although not selected here as a basis for the RPF derivation, *CYP7A1* was downregulated by PFOS in the present study (Figure 5). Reduction of *CYP7A1* expression has been observed before in HepaRG cells (Behr et al., 2020, Louisse et al., 2020), but also in human hepatocytes (Beggs et al. 2016) after exposure to PFASs. Currently, an AOP is under development on ‘HNF4alpha suppression leading to hepatic steatosis’ (access AOP-wiki 18-07-2022).

***ATF4, SLC7A11, YARS1***

Upon endoplasmic reticulum stress and/or amino acid starvation (Harding et al., 2000), genes that play a role in cell recovery, adaptation to stress conditions, and restoration of cell homeostasis are upregulated by the transcription factor ATF4 (Rozpedek et al., 2016). *SLC7A11* and *YARS1* have been described to be regulated by ATF4 (Adams, 2007; Shan et al., 2016; Krokowski et al., 2013; Han et al., 2013). Various of the PFASs tested in the present study caused an increase in the expression of *ATF4*, *SLC7A11* and/or *YARS1*. The *SLC7A11* gene codes for an amino acid transporter, and the *YARS1* gene codes for the aminoacyl-tRNA synthetase (ARS) that catalyzes the aminoacylation of tRNA with tyrosine. Although these processes may not directly play a role in liver toxicity, they may point to (PFAS-induced) endoplasmic reticulum stress and/or amino acid starvation that can be related to adverse effects to the liver. PFOA-induced endoplasmic reticulum stress in the liver has been reported in vivo in mice, and related liver toxicity was attenuated by the endoplasmic reticulum stress inhibitor 4-phenylbutyrate (Yan et al., 2015). Also other studies point to a possible role of endoplasmic reticulum stress in liver disease (Malhi and Kaufman, 2011; Maiers and Malhi, 2019).

***PDK4* and *ANGPTL4***

*PDK4* and *ANGPTL4* are PPARα-response genes, where it must be noted that *PDK4* expression is also regulated by retinoic acid receptors (Kwon and Harris, 2004). The present study shows that the PFCAs and PFSAs up to C10/C11 are activators of *PDK4* and *ANGPTL4* expression, whereas the longer chain PFASs (PFDoDA, PFTeDA, PFHxDA, PFODA and PFDS) are not. PPARα activity of the shorter chain PFASs has been reported before by Behr et al. (2020), whereas they did not test the effects of the longer chain PFAS that were not active in our study. Interestingly, the long-chain PFASs have been reported to be rather potent in vivo with regard to liver toxicity, which may suggest that liver toxicity is not necessarily related to PPARα activation. Although PPARα activation has been suggested to play a role in certain adverse effects to the liver, it is not typically related to the relative liver weight increase which was the endpoint used for RPF derivation based on in vivo studies (Bil et al., 2021; 2022a; 2022b; Hall et al., 2012).

***HMGCR* and *LSS***

*HMGCR* and *LSS* are cholesterogenic genes that were already found to be downregulated by PFOA, PFOS and PFNA in our previous study (Louisse et al., 2020). Various human (epidemiological) studies have reported a relationship between PFOA/PFOS (internal) exposure and increased cholesterol levels, but the causality is debated, and an established mode of action supporting such a relationship is lacking (Fragki et al., 2021). The downregulation of these genes found in HepaRG cells may at first seem better in line with the reported PFOA-induced decrease in cholesterol as observed in late-stage cancer patients in a phase I dose-escalation trial exposed to high doses of PFOA for six weeks (Convertino et al., 2018) as well as the PFAS-induced decrease in cholesterol in several animal studies (see review in Fragki et al. (2021)). On the other hand, suppressed *de novo* biosynthesis of cholesterol may point to an increase in intrahepatic cholesterol (Brown and Goldstein, 1997; Feingold et al. 2000; DeBose-Boyd, 2008). It is of importance to note that processes involved in regulation of cholesterol homeostasis in vivo are complex, and extrapolation of findings from a single cell model in vitro to consequences for in the in vivo situation is not straightforward.

***CXCL10***

CXCL10 may play a role in liver disease, but this is reported to be related to interaction with the immune system, playing a role in inflammation reactions upon liver damage (Chen et al., 2013). The impact of a decrease in *CXCL10* gene expression in liver cells upon PFAS exposure as observed in the present study on liver function is not known. It is, however, tempting to speculate about a possible role of a decrease in CXCL10 on the immune system, as an immunotoxic effect (decreased response to vaccination) has been selected as the critical endpoint for derivation of the health based guidance value of combined exposure to PFOA, PFNA, PFHxS and PFOS by EFSA (EFSA CONTAM Panel, 2020). CXCL10 is a chemokine playing a role in the stimulation and migration of immune cells and the regulation of T-cell and bone marrow progenitor maturation (Neville et al., 1997). PFOA and PFOS were shown before to decrease *CXCL10* gene expression in primary human liver cells (Beggs et al., 2016) and to decrease CXCL10 excretion by human bronchial epithelial cells (HBEC3-KT cell line) (Sørli et al., 2020). CXCL10 has been shown to work as an adjuvant, i.e., triggering an immune response to injected antigens in a mouse model (Krathwohl and Anderson, 2006). It is therefore tempting to speculate on a possible role of PFAS-induced CXCL10 suppression in the decreased vaccine response in humans (Grandjean et al., 2012; Abraham et al., 2020) and the decreased T-cell-dependent antibody response (TDAR) in experimental animals (Peden Adams et al., 2008; DeWitt et al., 2008; DeWitt et al., 2009; Dong et al., 2009), given that the mode of action underlying the immunotoxicity of PFASs has not been unraveled yet. CXCL10 has been shown to be directly involved in the generation of a parasite specific CD8+ T cell-mediated immune response in mice, evidenced by a significant reduction of CD8+ T cells in mice depleted of CXCL10 (Majumder et al., 2012). A PFAS-induced decrease in CXCL10 has not been reported in vivo, so any role in the immunotoxicity of PFASs is to be further investigated.

***THRSP***

THRSP has been reported to be involved in the regulation of lipid metabolism. It is induced by thyroid hormone, progestin, glucose and estradiol (Kuemmerle and Kinlaw, 2011: Ren et al., 2017). Expression has been reported to be downregulated upon fasting. A role of THRSP in PFAS-induced hepatotoxicity has not been described. Thrsp was up-regulated in livers of two mouse models (db/db mice and high-fat-diet-fed mice) for studying nonalcoholic fatty liver disease pathogenesis (Wu et al., 2013). This study also showed that hepatic overexpression of Thrsp increased triglyceride accumulation with enhanced lipogenesis in livers of C57Bl/6 mice, and that hepatic Thrsp gene silencing attenuated the fatty liver phenotype in db/db mice. THRSP expression has been reported to be regulated via various transcription factors, such as TH, PXR and CAR. Our previous study, compared the expression changes induced by PFOA, PFNA and PFOS in HepaRG cells with data published by Wigger et al. (2019) on the LXR agonist GW3965. Whereas the PFASs decreased *THRSP* expression, the LXR agonist GW3965 increased *THRSP* expression. Also various other genes induced by GW3965 were downregulated by the PFASs, suggesting a PFAS-induced downregulation of LXR-regulated genes. LXR activation has been described as the molecular initiating event in an AOP for liver steatosis (Vinken, 2015). A a role of inhibition of LXR-mediated gene expression in chemical-induced liver toxicity has not been described.

**References**

Abraham K, Mielke H, Fromme H, Völkel W, Menzel J, Peiser M, Zepp F, Willich SN, Weikert C (2020). Internal exposure to perfluoroalkyl substances (PFASs) and biological markers in 101 healthy 1-year-old children: associations between levels of perfluorooctanoic acid (PFOA) and vaccine response. Arch Toxicol. 94(6): 2131-2147.

Adams CM (2007). Role of the transcription factor ATF4 in the anabolic actions of insulin and the anti-anabolic actions of glucocorticoids. J Biol Chem. 282(23): 16744-16753.

Beggs KM, McGreal SR, McCarthy A, Gunewardena S, Lampe JN, Lau C, Apte U (2016). The role of hepatocyte nuclear factor 4-alpha in perfluorooctanoic acid- and perfluorooctanesulfonic acid-induced hepatocellular dysfunction. Toxicol Appl Pharmacol. 304: 18-29.

Behr AC, Plinsch C, Braeuning A, Buhrke T (2020). Activation of human nuclear receptors by perfluoroalkylated substances (PFAS). Toxicol In Vitro. 62: 104700.

Bil W, Zeilmaker M, Fragki S, Lijzen J, Verbruggen E, Bokkers B (2021). Risk Assessment of Per- and Polyfluoroalkyl Substance Mixtures: A Relative Potency Factor Approach. Environ Toxicol Chem. 40(3): 859-870.

Bil W, Zeilmaker M, Fragki S, Lijzen J, Verbruggen E, Bokkers B (2022). Response to Letter to the Editor on Bil et al. 2021 "Risk Assessment of Per- and Polyfluoroalkyl Substance Mixtures: A Relative Potency Factor Approach". Environ Toxicol Chem. 41(1): 13-18.

Bil W, Zeilmaker MJ, Bokkers BGH (2022b). Internal Relative Potency Factors for the Risk Assessment of Mixtures of Per- and Polyfluoroalkyl Substances (PFAS) in Human Biomonitoring. Environ Health Perspect. 130(7):77005.Brown MS, Goldstein JL (1997). The SREBP pathway: regulation of cholesterol metabolism by proteolysis of a membrane-bound transcription factor. Cell. 89(3): 331-340.

Chen LJ, Lv J, Wen XY, Niu JQ (2013). CXC chemokine IP-10: a key actor in liver disease? Hepatol Int. 7(3): 798-804.

Convertino M, Church TR, Olsen GW, Liu Y, Doyle E, Elcombe CR, Barnett AL, Samuel LM, MacPherson IR, Evans TRJ (2018). Stochastic Pharmacokinetic-Pharmacodynamic Modeling for Assessing the Systemic Health Risk of Perfluorooctanoate (PFOA). Toxicol Sci. 163(1): 293-306.

DeBose-Boyd RA (2008). Feedback regulation of cholesterol synthesis: sterol-accelerated ubiquitination and degradation of HMG CoA reductase. Cell Res. 18(6): 609-621.

Dewitt JC, Copeland CB, Strynar MJ, Luebke RW (2008). Perfluorooctanoic acid-induced immunomodulation in adult C57BL/6J or C57BL/6N female mice. Environ Health Perspect. 116(5): 644-650.

DeWitt JC, Copeland CB, Luebke RW (2009). Suppression of humoral immunity by perfluorooctanoic acid is independent of elevated serum corticosterone concentration in mice. Toxicol Sci. 109(1): 106-112.

Dong GH, Zhang YH, Zheng L, Liu W, Jin YH, He QC (2009). Chronic effects of perfluorooctanesulfonate exposure on immunotoxicity in adult male C57BL/6 mice. Archives of Toxicology, 83, 805–815.

Eeckhoute J, Formstecher P, Laine B (2004). Hepatocyte Nuclear Factor 4α enhances the Hepatocyte Nuclear Factor 1α‐mediated activation of transcription. Nucleic Acids Research 32(8): 2586-2593.

EFSA CONTAM Panel (2020). Scientific Opinion. Risk to human health related to the presence of perfluoroalkyl substances in food. EFSA Journal 18 (9):6223.

Feingold KR. 2000. Introduction to lipids and lipoproteins. In: Feingold KR, Anawalt B, Boyce A, Chrousos G, de Herder WW, Dungan K, Grossman A, Hershman JM, Hofland J, Kaltsas G, editors. Endotext [Internet]. South Dartmouth (MA): MDText.com. [Updated 2018 Feb 2]. Available from: https://www.ncbi.nlm.nih.gov/books/NBK305896/.

Fragki S, Dirven H, Fletcher T, Grasl-Kraupp B, Bjerve Gützkow K, Hoogenboom R, Kersten S, Lindeman B, Louisse J, Peijnenburg A, Piersma AH, Princen HMG, Uhl M, Westerhout J, Zeilmaker MJ, Luijten M (2021). Systemic PFOS and PFOA exposure and disturbed lipid homeostasis in humans: what do we know and what not? Crit Rev Toxicol. 51(2): 141-164.

Grandjean P, Andersen EW, Budtz-Jørgensen E, Nielsen F, Mølbak K, Weihe P, Heilmann C (2012). Serum vaccine antibody concentrations in children exposed to perfluorinated compounds. JAMA. 307(4): 391-397.

Hall AP, Elcombe CR, Foster JR, Harada T, Kaufmann W, Knippel A, Küttler K, Malarkey DE, Maronpot RR, Nishikawa A, Nolte T, Schulte A, Strauss V, York MJ (2012). Liver hypertrophy: a review of adaptive (adverse and non-adverse) changes - conclusions from the 3rd International ESTP Expert Workshop. Toxicol Pathol. 40(7): 971-994.

Han J, Back SH, Hur J, Lin YH, Gildersleeve R, Shan J, Yuan CL, Krokowski D, Wang S, Hatzoglou M, Kilberg MS, Sartor MA, Kaufman RJ (2013). ER-stress-induced transcriptional regulation increases protein synthesis leading to cell death. Nat Cell Biol. 15: 481-490.

Harding HP, Novoa I, Zhang Y, Zeng H, Wek R, Schapira M, Ron D (2000). Regulated translation initiation controls stress-induced gene expression in mammalian cells. Mol Cell. 6(5): 1099-1108.

Klein K, Jüngst C, Mwinyi J, Stieger B, Krempler F, Patsch W, Eloranta JJ, Kullak-Ublick GA (2010). The human organic anion transporter genes OAT5 and OAT7 are transactivated by hepatocyte nuclear factor-1α (HNF-1α). Mol Pharmacol. 78(6): 1079-1087.

Krathwohl MD, Anderson JL (2006). Chemokine CXCL10 (IP-10) is sufficient to trigger an immune response to injected antigens in a mouse model. Vaccine. 24(15): 2987-2993.

Krokowski D, Han J, Saikia M, Majumder M, Yuan CL, Guan BJ, Bevilacqua E, Bussolati O, Bröer S, Arvan P, Tchórzewski M, Snider MD, Puchowicz M, Croniger CM, Kimball SR, Pan T, Koromilas AE, Kaufman RJ, Hatzoglou M (2013). A self-defeating anabolic program leads to β-cell apoptosis in endoplasmic reticulum stress-induced diabetes via regulation of amino acid flux. J Biol Chem. 288: 17202-17213.

Kuemmerle NB, Kinlaw WB (2011). THRSP (thyroid hormone responsive). Atlas Genet Cytogenet Oncol Haematol. 15(6): 480-482.

Kwon HS, Harris RA (2004). Mechanisms responsible for regulation of pyruvate dehydrogenase kinase 4 gene expression. Adv Enzyme Regul. 44: 109-121.

Louisse J, Rijkers D, Stoopen G, Janssen A, Staats M, Hoogenboom R, Kersten S, Peijnenburg A (2020). Perfluorooctanoic acid (PFOA), perfluorooctane sulfonic acid (PFOS), and perfluorononanoic acid (PFNA) increase triglyceride levels and decrease cholesterogenic gene expression in human HepaRG liver cells. Arch Toxicol. 94(9): 3137-3155.

Maiers JL, Malhi H (2019). Endoplasmic Reticulum Stress in Metabolic Liver Diseases and Hepatic Fibrosis. Semin Liver Dis. 39(2): 235-248.

Majumder S, Bhattacharjee S, Paul Chowdhury B, Majumdar S (2012). CXCL10 is critical for the generation of protective CD8 T cell response induced by antigen pulsed CpG-ODN activated dendritic cells. PLoS One. 7(11): e48727.

Malhi H, Kaufman RJ (2011). Endoplasmic reticulum stress in liver disease. J Hepatol. 54(4): 795-809.

Miao J, Fang S, Bae Y, Kemper JK (2006). Functional inhibitory cross-talk between constitutive androstane receptor and hepatic nuclear factor-4 in hepatic lipid/glucose metabolism is mediated by competition for binding to the DR1 motif and to the common coactivators, GRIP-1 and PGC-1alpha. J Biol Chem. 281(21): 14537-14546.

Neville LF, Mathiak G, Bagasra O (1997). The immunobiology of interferon-gamma inducible protein 10 kD (IP-10): a novel, pleiotropic member of the C-X-C chemokine superfamily. Cytokine Growth Factor Rev. 8(3): 207-219.

Peden-Adams MM, Keller JM, Eudaly JG, Berger J, Gilkeson GS, Keil DE (2008). Suppression of humoral immunity in mice following exposure to perfluorooctane sulfonate. Toxicol Sci. 104: 144–154.

Ren J, Xu N, Zheng H, Tian W, Li H, Li Z, Wang Y, Tian Y, Kang X, Liu X (2017). Expression of Thyroid Hormone Responsive SPOT 14 Gene Is Regulated by Estrogen in Chicken (Gallus gallus). Sci Rep. 7(1): 10243.

Rozpedek W, Pytel D, Mucha B, Leszczynska H, Diehl JA, Majsterek I (2016). The Role of the PERK/eIF2α/ATF4/CHOP Signaling Pathway in Tumor Progression During Endoplasmic Reticulum Stress. Curr Mol Med. 16: 533-544.

Shan J, Zhang F, Sharkey J, Tang TA, Örd T, Kilberg MS (2016). The C/ebp-Atf response element (CARE) location reveals two distinct Atf4-dependent, elongation-mediated mechanisms for transcriptional induction of aminoacyl-tRNA synthetase genes in response to amino acid limitation. Nucleic Acids Res. 44: 9719-9732.

Shih DQ, Bussen M, Sehayek E, Ananthanarayanan M, Shneider BL, Suchy FJ, Shefer S, Bollileni JS, Gonzalez FJ, Breslow JL, Stoffel M (2001). Hepatocyte nuclear factor-1alpha is an essential regulator of bile acid and plasma cholesterol metabolism. Nat Genet. 27(4): 375-382.

Sørli JB, Låg M, Ekeren L, Perez-Gil J, Haug LS, Da Silva E, Matrod MN, Gützkow KB, Lindeman B (2020). Per- and polyfluoroalkyl substances (PFASs) modify lung surfactant function and pro-inflammatory responses in human bronchial epithelial cells. Toxicol In Vitro. 62: 104656.

Vinken M (2015). Adverse Outcome Pathways and Drug-Induced Liver Injury Testing. Chem Res Toxicol. 28(7): 1391-1397.

Wigger L, Casals-Casas C, Baruchet M, Trang KB, Pradervand A, Naldi A, Desvergne B (2019). System analysis of cross-talk between nuclear receptors reveals an opposite regulation of the cell cycle by LXR and FXR in human HepaRG liver cells. PLoS One. 14: e0220894.

Wu J, Wang C, Li S, Li S, Wang W, Li J, Chi Y, Yang H, Kong X, Zhou Y, Dong C, Wang F, Xu G, Yang J, Gustafsson JÅ, Guan Y (2013). Thyroid hormone-responsive SPOT 14 homolog promotes hepatic lipogenesis, and its expression is regulated by liver X receptor α through a sterol regulatory element-binding protein 1c-dependent mechanism in mice. Hepatology. 58(2): 617-628.

Yan S, Zhang H, Wang J, Zheng F, Dai J (2015). Perfluorooctanoic acid exposure induces endoplasmic reticulum stress in the liver and its effects are ameliorated by 4-phenylbutyrate. Free Radic Biol Med. 87: 300-311.
